# Analyses of the essential C82 subunit uncovered some differences in RNA polymerase III transcription between *Trypanosoma brucei* and *Leishmania major*

**DOI:** 10.1017/S0031182024000921

**Published:** 2024-09

**Authors:** Andrés Cano-Santiago, Luis E. Florencio-Martínez, Daniel E. Vélez-Ramírez, Adrián J. Romero-Chaveste, Rebeca G. Manning-Cela, Tomás Nepomuceno-Mejía, Santiago Martínez-Calvillo

**Affiliations:** 1 Unidad de Biomedicina, Facultad de Estudios Superiores Iztacala, Universidad Nacional Autónoma de México, Tlalnepantla, México; 2 Departamento de Biomedicina Molecular, Centro de Investigación y de Estudios Avanzados del IPN, Ciudad de México, México

**Keywords:** *Leishmania major*, RNAP III transcription, C82, tRNA, *Trypanosoma brucei*, 5S rRNA

## Abstract

The 17-subunit RNA polymerase III (RNAP III) synthesizes essential untranslated RNAs such as tRNAs and 5S rRNA. In yeast and vertebrates, subunit C82 forms a stable subcomplex with C34 and C31 that is necessary for promoter-specific transcription initiation. Little is known about RNAP III transcription in trypanosomatid parasites. To narrow this knowledge gap, we characterized the C82 subunit in *Trypanosoma brucei* and *Leishmania major*. Bioinformatic analyses showed that the 4 distinctive extended winged-helix (eWH) domains and the coiled-coil motif are present in C82 in these microorganisms. Nevertheless, C82 in trypanosomatids presents certain unique traits, including an exclusive loop within the eWH1 domain. We found that C82 localizes to the nucleus and binds to RNAP III-dependent genes in the insect stages of both parasites. Knock-down of C82 by RNA interference significantly reduced the levels of tRNAs and 5S rRNA and led to the death of procyclic forms of *T. brucei*. Tandem affinity purifications with both parasites allowed the identification of several C82-interacting partners, including C34 and some genus-specific putative regulators of transcription. However, the orthologue of C31 was not found in trypanosomatids. Interestingly, our data suggest a strong association of C82 with TFIIIC subunits in *T. brucei*, but not in *L. major*.

## Graphical abstract

**Figure:**
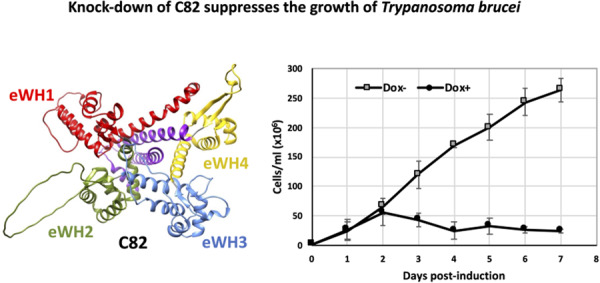

## Introduction

In all organisms, RNA polymerases (RNAP) are multimeric enzymes responsible for synthesizing RNA molecules. In eukaryotes, nuclear genomes are transcribed by DNA-dependent RNAP I, II and III (Roeder, 2019). RNAP I produces ribosomal RNAs (rRNAs) 18S, 5.8S and 28S, whereas RNAP II transcribes multiple types of RNA molecules, including messenger RNAs (mRNAs), most small nuclear RNAs (snRNAs) and small nucleolar RNAs (snoRNAs) (Liu *et al*., 2013). RNAP III mediates the synthesis of structured, small RNAs such as transfer RNAs (tRNAs), 5S rRNA, U6 snRNA and 7SL RNA, many of which have functions related to protein synthesis and RNA processing (Dieci *et al*., 2013).

In yeast and vertebrates, most RNAP III promoters consist of sequence elements located downstream of the transcription start sites within the transcribed region, and they are classified into 3 main classes (Dieci *et al*., 2007; Leśniewska and Boguta, 2017). Type I promoters are typical of 5S rRNA genes, and they consist of 3 internal domains: Box A, an intermediate element and Box C. tRNA genes contain type II promoters that possess 2 conserved internal elements, Boxes A and B. Type III promoters, characteristic of U6 snRNA genes, consist of elements that reside exclusively upstream of the coding sequence: a TATA box, a proximal sequence element and a distal sequence element (Dieci *et al*., 2013).

General transcription factors TFIIIA, TFIIIB and TFIIIC are required by RNAP III to initiate RNA synthesis (Geiduschek and Kassavetis, 2001). While TFIIIA is a single-subunit factor involved exclusively in the 5S rRNA production, TFIIIB participates in the transcription of all RNAP III-dependent genes; it is composed of the TATA-binding protein (TBP), the TFIIB-related factor 1 (Brf1) and B double prime 1 (Bdp1). TFIIIC is a 6-protein complex required for tRNA and 5S rRNA transcription. Another protein that regulates RNAP III activity is the transcriptional repressor Maf1 (Graczyk *et al*., 2018).

RNAP III is the largest of all eukaryotic RNAP, as it is composed of 17 different subunits (in comparison to 12 and 14 subunits for RNAP II and I, respectively) with a molecular weight of 0.7 MDa (Girbig *et al*., 2021). Five subunits are shared between all 3 RNAPs: RPB5, RPB6, RPB8, RPB10 and RPB12. Subunits AC40 and AC19 are common to RNAP I and III, and another 5 subunits (C160, C128, C25, C17 and C11) are homologous to RNAP I and II subunits. The remaining 5 subunits are specific to RNAP III, as they do not have structurally equivalent subunits in RNAP I or RNAP II. They form 2 stable subcomplexes, the C82/C34/C31 heterotrimer and the C53/C37 heterodimer (Geiduschek and Kassavetis, 2001; Hu *et al*., 2002).

The C82/C34/C31 heterotrimer is critical for specific transcription initiation at RNAP III-dependent genes, as it contributes to promoter opening (Wang and Roeder, 1997). Moreover, the C82/C34/C31 subcomplex seems to participate in the stabilization of the transcription bubble during RNA elongation (Lefèvre *et al*., 2011). C82, also known as RPC82 or RPC3 (and as RPC62 in humans), is characterized by the presence of 4 extended winged-helix (eWH) domains and a C-terminal coiled-coil domain. The eWH regions are required for the interactions that C82 establishes not only with C34 and C31, but also with C160 and C128, and with the Brf1 subunit of TFIIIB (Boissier *et al*., 2015; Hoffmann *et al*., 2015; Khoo *et al*., 2018). C82 also binds to promoter regions (Lefèvre *et al*., 2011; Khoo *et al*., 2018; Vorländer *et al*., 2018), and displays helicase activity to unwind double-stranded DNA in an ATP-dependent fashion (Ayoubi *et al*., 2019). Thus, C82 plays key roles in RNAP III preinitiation complex formation and transcription initiation and elongation.

The parasitic protozoa *Trypanosoma brucei* and *Leishmania* spp. are members of the trypanosomatid family that affect millions of people worldwide. *Trypanosoma brucei* is endemic to Sub-Saharan Africa, where it produces sleeping sickness, also called human African trypanosomiasis (Pays *et al*., 2023). The parasite is transmitted to humans by tsetse flies of the *Glossina* genus. Several species of *Leishmania* produce different types of leishmaniases in around 98 countries on 5 continents (Alvar *et al*., 2012; Singh *et al*., 2023). The pathogen is spread to humans by the bite of infected female sandflies of the genera *Phlebotomus* and *Lutzomyia*. Notably, trypanosomatids exhibit gene expression processes that are atypical among eukaryotes, such as RNAP II polycistronic transcription and mRNA maturation by trans-splicing (Martínez-Calvillo *et al*., 2010; Clayton, 2019).

In trypanosomatids, the knowledge about RNAP III transcription is scarce. Except for C31, orthologues of all RNAP III subunits have been identified in these parasites by tandem affinity purifications (Martínez-Calvillo *et al*., 2007; Florencio-Martínez *et al*., 2021) or *in silico* analyses (El-Sayed *et al*., 2005; Kelly *et al*., 2005; Das *et al*., 2008). Unlike other eukaryotes, trypanosomatid RNAP III transcribes not only the U6 snRNA gene, but the whole set of snRNA genes (Fantoni *et al*., 1994). Interestingly, Boxes A and B located within neighbouring tRNA genes control the expression of snRNA genes in *T. brucei* and *L. major* (Nakaar *et al*., 1997; Rojas-Sánchez *et al*., 2016). Regarding general transcription factors, subunits TBP, Brf1 and Bdp1 from TFIIIB are essential proteins required for transcription initiation of RNAP III-dependent genes in *L. major* and *T. brucei* (Thomas *et al*., 2006; Vélez-Ramírez *et al*., 2015; Román-Carraro *et al*., 2019; Florencio-Martínez *et al*., 2021). Also, a 4-subunit TFIIIC complex that associates with tRNA and U2 snRNA genes was recently identified in trypanosomatids (Mondragón-Rosas *et al*., 2024). The transcriptional repressor Maf1 (Romero-Meza *et al*., 2017) and the SNAP50 subunit of the SNAP complex (Thomas *et al*., 2009) have also been implicated in the regulation of RNAP III transcription in trypanosomatids.

In this work, we studied the C82 subunit of RNAP III in *T. brucei* (TbC82) and *L. major* (LmC82). While TbC82 and LmC82 possess the characteristic domains found in other C82 orthologues, they contain some unique features, including a trypanosomatid-specific loop within the eWH1 domain. Depletion of TbC82 by RNAi led to the growth arrest of the parasite, demonstrating that TbC82 is essential for *T. brucei* viability. Putative interacting partners of C82 were identified by tandem affinity purifications and mass spectrometry analyses, revealing similarities and differences between trypanosomatids and other organisms. Interestingly, some differences were also found between *T. brucei* and *L. major*.

## Materials and methods

### *In silico* analyses

Protein sequences were obtained from the TriTrypDB database (release 62) (http://tritrypdb.org/tritrypdb/) and the NCBI database (http://www.ncbi.nlm.nih.gov). Sequence alignments were made with the ClustalΩ program (http://www.ebi.ac.uk/Tools/msa/clustalo/) and shaded manually. Domain identification and secondary structure predictions were generated with the PSIPRED server (http://bioinf.cs.ucl.ac.uk/psipred/) and the Phyre2 program (http://www.sbg.bio.ic.ac.uk/~phyre2). The predicted 3-dimensional structures were generated with the AlphaFold program (https://alphafold.ebi.ac.uk/). Models were visualized and edited with the UCFS Chimera package (https://www.cgl.ucsf.edu/chimera/). Comparisons of the predicted structures were carried out with the iCn3D program (https://www.nlm.nih.gov/ncbi/workshops/2023-03_3d-molecular-structures/icn3d_alphafold.html). Hypothetical proteins identified by mass spectrometry were analysed with the HHpred program (https://toolkit.tuebingen.mpg.de/tools/hhpred), and the DALI server (http://ekhidna2.biocenter.helsinki.fi/dali/).

### Cell culture and electroporation of *L. major* and *T. brucei*

Promastigotes from *L. major* strain MHOM/IL/81/Friedlin (LSB-132.1) were grown in BM medium supplemented with 10% fetal bovine serum (Life Technologies Corporation, Grand Island, NY, USA) at 28°C (Florencio-Martínez *et al*., 2021). Transfection with the episomal pLmC82-PTP vector was performed by electroporation as previously described (Florencio-Martínez *et al*., 2021). Clones were obtained by spreading transfected cells on plates containing 0.7% Seaplaque GTG agarose (FMC Bioproducts, Philadelphia, PA, USA) in BM medium with 50 μg mL^−1^ G418.

Procyclic forms of *T. brucei* strain 29-13 (Wirtz *et al*., 1999) were cultured at 28°C in SDM-79 medium supplemented with 10% fetal bovine serum (Life Technologies Corporation), 50 μg mL^−1^ hygromycin B (Sigma-Aldrich, Darmstadt, Germany) and 15 μg mL^−1^ G418 (Sigma-Aldrich). Cells were transfected by electroporation, as previously described (Vélez-Ramírez *et al*., 2015). Populations were selected with phleomycin (2.5 μg mL^−1^) for RNAi or with blasticidin (10 μg mL^−1^) for PTP (Prot C-TEV-Prot A) tagging (Schimanski *et al*., 2005). Clones were obtained by serial dilution in 96-well plates. RNAi induction was carried out by adding doxycycline (2 μg mL^−1^) to the medium. The same clone was grown in the absence of doxycycline (non-induced control). For growth curves, parasites were counted daily and diluted to 2 × 10^6^ cells mL^−1^, and cumulative cell density was plotted.

### Generation of plasmids

To obtain the pC-TbC82-PTP plasmid for PTP-tagging, a 725-bp fragment from the C-terminal end of the TbC82 gene (Tb927.2.2990) was amplified with primers TbC82-C-PTP-ApaI-5’ (5’-GGGCCCTAGCGCATCAATGCCGCTT) and TbC82-C-PTP-NotI-3’-GC (5’-GCGGCCGCGCGTAAAAGTCCAAAACTAG). The DNA fragment was cloned into the genome-integration pC-PTP-BLA plasmid (Schimanski *et al*., 2005) with the *Apa*I and *Not*I restriction sites. The vector was linearized with the *Xcm*I enzyme before transfection. To produce the pLmC82-PTP vector, the complete LmC82 gene (LmjF.27.2600), without the terminal codon, was amplified by PCR with oligonucleotides C82-AgeI-5’ (5’-ATACCGGTATATTTCTCCTCAGCAGGACTC) and C82-XbaI-3’ (5’-ATTCTAGAAAAAAAATCAACAATCAGCAGC). The PCR product was cloned into the episomal pB6-PTP plasmid (Moreno-Campos *et al*., 2016) digested with *Xma*I and *Xba*I. To obtain plasmid p2T7-TbC82 for RNAi assays, a 419-bp fragment from the TbC82 gene was amplified with primers TbC82-RNAi-F (5’-AGGATCCAAGCTTGAACAACTCAGCCCTCTT) and TbC82-RNAi-R (5’-ACTCGAGATTGTCACACCCGTCTCTCC) and cloned into the p2T7-177 vector (Wickstead *et al*., 2002) digested with *Xho*I and *Bam*HI. Prior to transfection the vector was linearized with *Not*I. To produce plasmid pCold-TbC82, the entire TbC82 gene was amplified with primers TbC82-BamHI-F (5’-GGATCCATGCCACGGCGTGCTGAG) and TbC82-XbaI-R (5’-TCTAGAGTAAAAGTCCAAAACTAGC) and cloned into the *Bam*HI and *Xba*I restriction sites of the pCold1 expression vector (Takara Bio Inc., San Jose, CA, USA). All vectors were verified by sequencing.

### Western blot analysis

Whole-cell protein extracts were made as described previously (Florencio-Martínez *et al*., 2021). To perform Western blots, 20 μg of protein were fractionated by 10% SDS-PAGE and blotted onto PVDF (polyvinylidene difluoride membranes (Bio-Rad, Hercules, CA, USA). Next, the membranes were incubated with rabbit primary monoclonal anti-Prot C antibodies (Delta Biolabs, Boise, ID, USA) with a 1:3000 dilution, or polyclonal *β*-tubulin antibody (Thermo Scientific, Waltham, MA, USA) with a 1:1500 dilution; and then with a horseradish peroxidase (HRP)-conjugated secondary antibody with a 1:4000 dilution and developed with the Immobilon Western Chemiluminescent HRP substrate (Merck-Millipore, Darmstadt, Germany). The detection of TbC82 was achieved with a polyclonal anti-TbC82 antiserum (with a 1:500 dilution).

### Indirect immunofluorescence

The subcellular localization of the TbC82-PTP and LmC82-PTP proteins was analysed by indirect immunofluorescence assays as previously described (Nepomuceno-Mejía *et al*., 2018). To detect TbC82-PTP, *T. brucei* cells were fixed with 4% paraformaldehyde and incubated with rabbit anti-Prot C antibody (Delta Biolabs) followed by secondary anti-rabbit antibody conjugated with Alexa-Fluor 488 (Life Technologies Corporation). Likewise, *L. major* cells were fixed with 4% paraformaldehyde and incubated with a rabbit anti-Prot C antibody. Then, a mouse anti-LmNop56 antibody was used as a nucleolar marker. Parasites were then treated with a mixture of secondary anti-rabbit antibody conjugated with Alexa-Fluor 488 and anti-mouse antibody conjugated with Alexa Fluor 568 (Life Technologies Corporation). DNA was stained with DAPI (4′,6-diamidine-2′-phenylindole dihydrochloride). Images were obtained with a Zeiss AxioImager A2 microscope and analysed with the ZEN 2012 software (Blue 217 edition) (Zeiss, Oberkochen, Germany).

### Northern blot analysis

The abundance of the TbC82 mRNA after RNAi was analysed by Northern blot experiments. For that purpose, total RNA was extracted with the TRI reagent (Sigma-Aldrich), and 20 μg were run in an agarose-formaldehyde denaturing gel and transferred to Hybond-N nylon membrane (GE HealthCare, Chicago, IL, USA). The radioactive probe employed corresponded to the 419-bp fragment cloned into the p2T7-TbC82 plasmid, labelled with [*α*-32P]-dCTP using the High Prime DNA Labelling Kit (Roche, Basel, Switzerland). Membranes were hybridized with a solution of formamide 50%, saline-sodium citrate (SSC) buffer 5×, sodium dodecyl sulphate (SDS) 0.2%, Denhardts 4× and salmon sperm DNA (100 μg mL^−1^) at 42°C, and then washed to a final stringency of 0.1 × SSC and 0.1% SDS at 65°C.

### Tandem affinity purifications and mass spectrometry analysis

The proteins that associate with TbC82 and LmC82 were determined by performing tandem affinity purification assays, in duplicate, with parasites from the TbC82-PTP and LmC82-PTP cell lines (3 L at 2–3 × 10^7^ cells mL^−1^) as previously described (Florencio-Martínez *et al*., 2021). After the second column, the eluted proteins were concentrated with Amicon Ultra 3 K columns (Merck-Millipore) and by evaporation in a vacuum concentrator. Proteins were then analysed by SDS–PAGE and SYPRO Ruby (Invitrogen, Carlsbad, CA, USA) staining. Individual lanes from the gels were sliced into 2 pieces and proteins subjected to in-gel tryptic digestion prior to liquid chromatography–mass spectrometry/mass spectrometry at the Core Facility for Proteomics and Mass Spectrometry from Upstate Medical University (Syracuse, NY, USA). The collision-induced dissociation spectra were compared with the *T. brucei* and *L. major* protein database from the TriTrypDB page.

### Chromatin immunoprecipitation (ChIP) assays

ChIP assays were carried out 3 times with the TbC82-PTP and LmC82-PTP cell lines, as described previously (Romero-Meza *et al*., 2017). Briefly, 1.2 × 10^8^ cells were cross-linked with 1% formaldehyde for 5 min at 37°C, and lysed with a Vibra-Cell VCX130 ultrasonic processor (Sonics, Newtown, CT, USA) (15 s on/off, 40% amplitude, for 3 min). Nuclei were pelleted and resuspended in sonication buffer (1% SDS, 10 mm EDTA and 50 mm Tris-HCl, pH 8.0, with 1× protease inhibitors). Chromatin was sonicated with a BioRuptor UCD-200 (Diagenode, Denville, NJ, USA) (30 s on/30 s off, high intensity) for 40 cycles, to an average DNA size of around 200–500 bp. The sonicated material was pre-cleared with protein A/G plus-agarose beads (Santa Cruz Biotechnology, Dallas, TX, USA) by mixing for 1 h at 4°C. Chromatin samples were incubated overnight at 4°C with rabbit anti-Prot A antibody (Sigma-Aldrich) or non-specific rabbit serum as negative control. The protein–DNA complexes were incubated for 2 h with protein A/G plus-agarose beads and 20 μg of sonicated salmon sperm DNA, and then washed as previously described (Vizuet-de-Rueda *et al*., 2016). The cross-links were reversed with 200 mm NaCl at 65°C overnight and then treated with RNase A and proteinase K. DNA was precipitated with sodium acetate and ethanol and quantified.

### Quantitative PCR assays

Quantitative PCR (qPCR) was performed with 2 ng of immunoprecipitated DNA to identify the regions of DNA to which TbC82 and LmC82 bind. The reactions were performed in duplicate, using optimized primers and conditions that produce a single amplicon of the correct size, with the Platinum SYBR Green qPCR SuperMix-UDG kit (Invitrogen). Results were analysed with the 2^−ΔΔCq^ method, as reported before (Vélez-Ramírez *et al*., 2015; Vizuet-de-Rueda *et al*., 2016), and are presented as percentage of input, corrected by subtracting the corresponding values from negative control precipitations performed with a non-specific antiserum. The upstream control region of the rRNA promoter (18S rRNA prom) of *T. brucei* was amplified with primers 18SUSE5 (5’-CACCCTCAAGACCGTAGCTC) and 18SUSE3 (5’-ACCCGTCCCTTATCAACACA). The 18S rRNA gene (Tb927.2.1452) was amplified with oligonucleotides 18SqFw (5’-GGGATACTCAAACCCATCCA) and 18SqRv (5’-CCCTTTAACAGCAACAGCATTA); and the *α*-tubulin gene (Tb927.1.2340) with TubqFw (5’-GGGCTTCCTCGTGTATCA) and TubqRv (5’-GCTTGGACTTCTTGCCATAG). The promoter of the *SL* gene (SL prom) was amplified with oligonucleotides SL-promoter-F (5’-CTACCGACACATTTCTGGC) and SL-promoter-R (5’-GCTGCTACTGGGAGCTTCTCATACC). The 5S rRNA gene (Tb927.8.1381) was amplified with primers rRNA5S-5′ (5’-GTCGAGTACGACCACACTTG) and rRNA5S-3’ (5’-AAGAGTACGGCACTCAGGGT). The tRNA-Ala gene (Tb927.7.6821) was amplified with primers AlaqFw (5’-GGGGATGTAGCTCAGATGG) and AlaqRv (5’-TGGAGAAGTTGGGTATCGATC); and the tRNA-Arg gene (Tb927.8.2859) with primers ArgqFw (5’-GGTCTCGTGGCGCAATG) and ArgqRv (5’-CGATCCCGGCAGGACTC). The intergenic region upstream of the tRNA-Ala gene (tRNA Ala inter) was amplified with primers InterAla5’ (5’-CACTCTCCCGAGAATCGAAG) and InterAla3’ (5’-TGGGTGTGGAGTCGACTTTT). The U2 snRNA gene (Tb927.2.5680) was amplified with primers U2qFw (5’-CTCGGCTATTTAGCTAAGATCAAGT) and U2qRv (5’-CGGGACAGCCAACAGTTT); and its promoter region (U2 snRNA prom) with primers U2Prom5’ (5’-CACAACCTGTAGTGGCGGTA) and Tb-U2-R (5’-GCATATCTTCTCGGCTATT).

For *L. major*, the 18S rRNA promoter (18S rRNA prom) was amplified with oligonucleotides rRNA-A-5’ (5’-TTGTTTGGGTGGAGGTGAGA) and rRNA-A-3’ (5’-CAAAATCATCAAACCCGTTC); and the rRNA 18S gene was amplified with primers rRNA-18S-5’ (5’-CATGCATGCCTCAGAATCAC) and rRNA-18S-3’ (5’-CGTTTCGCCAAGTTATCCAA). The protein-coding gene LmjF.11.0930 was amplified with primers 11.0930-5’ (5’-AGCAGCAGTTCATTGAGGCT) and 11.0930-3’ (5’-GCCGATCATCATCCTCTAAG); and the intergenic region upstream of LmjF.11.0930 (Inter 11.0930) was analysed with oligonucleotides 11-Inter-5’ (5’-GAACTTGGGAATGCCTTCTG) and 11-Inter-3’ (5’-GCAAGAAGAATGTGGAACGG). The amplification of SL promoter (SL prom) was carried out with primers LmjF-SL-PromF (5’-GAGCGCGGTGGGCATGACA) and LmjF-SL-PromR (5’-AAGCCATCACCACCGCAGC); and the intergenic region of the *SL* gene (Inter SL) was amplified with oligonucleotides LmjF-SL-InterF (5’-TGTGCGTGCGTGTGGTGGT) and LmjF-SL-InterR (5’-CGGGCGCACCCTTGCAGT). The strand-switch region (SSR) of chromosome 1 (SSR Chr. 1) was amplified with primers Ssr4-F (5’-AATCACAGCACGCATACACG) and Ssr4-R (5’-GCGTCATGGCTTCACTAACAG). The 5S rRNA gene was amplified with oligonucleotides 5SrRNA-F1 (5’-GAGTACGACCACACTTGAGTG) and 5SrRNA-R1 (5’-GAGTACGGCACTCAGGGTT). The U2 snRNA was analysed with primers U2-5’ (5’-AAACGTGGAACTCCAAGGAA) and U2-3’ (5’-TATCTTCTCGGCTATTTAGC); and the promoter region of the U2 snRNA (U2 snRNA prom) with primers U2tRNA-like-5’ (5’-CCGAGAAGATATGTTAGTACCACC) and U2tRNA-like-3’ (5’-AGGAAAAGATGCTTTCGACGAG). The tRNA-Met gene was amplified with oligonucleotides tRNAmet-F (5’-AAAGTTTGCGACCGGTGAG) and tRNAmet-R (5’-CACAACTTTCACTCGTAGCCG).

### Reverse transcription qPCR

The abundance of different transcripts after TbC82 depletion by RNAi was analysed by reverse transcription qPCR (RT-qPCR) assays. Three biological replicates were examined. Briefly, 1 μg of total RNA from the induced and non-induced cultures was used as template for the first strand cDNA synthesis using the SuperScript III Reverse Transcriptase (Invitrogen) and 50 ng of random hexamers (Invitrogen). The cDNA was analysed by qPCR using the Platinum SYBR Green qPCR SuperMix-UDG kit (Invitrogen). The qPCR reactions were performed in duplicate. The procyclin transcript (Tb927.6.510) was amplified with primers Procyclin-5’ (5’-ATGGCACCTCGTTCCCTTTA) and ProcqRv (5’-CTTTGCCTCCCTTCACGATAAC); and TFIIB (Tb927.9.5710) with primers Tf2bqFw (5′-GAACAGGGAACGCACATTAG) and Tf2bqRv (5′-TTGTTGACTTTGGTCACTTCC). The 5S rRNA (Tb927.8.1381) was amplified with primers rRNA5S-5′ (5’-GTCGAGTACGACCACACTTG) and 5S rRNA-3’ (5’-TGAGCCTGTGAGTGCTTAACTT); and the TbC82 transcript was analysed with primers TbC82-GFP-F (5’-AGGTACCACCGGCTTCCAAAGAACT) and TbC82-C-PTP-NotI-3 (5’-GCGGCCGCGTAAAAGTCCAAAACTAGCATC). The *α*-tubulin, tRNA-Ala, tRNA-Arg and U2 snRNA were amplified with the primers mentioned in the previous section.

### Production of TbC82 polyclonal antibody

*Escherichia coli* BL21 (DE3) competent cells (Thermo Scientific) were transformed with the pCold-TbC82 plasmid. Induction of the TbC82 recombinant protein (TbC82r) expression was achieved with 1 mm isopropyl *β*-D-1-thiogalactopyranoside at 37°C for 18 h. The TbC82r protein was purified by affinity chromatography with Ni-Sepharose 6 Fast Flow matrix (GE Healthcare), according to the manufacturer's instructions. The anti-TbC82 polyclonal antibody was produced by inoculating 6-week-old male BALB/c mice intravenously with 100 μg of purified TbC82r protein mixed with TiterMax Gold adjuvant (Sigma-Aldrich) at a 1:1 ratio. Pre-immune mouse serum was obtained before antigen inoculation. Blood samples were collected 6 weeks after antigen immunization, and anti-TbC82 polyclonal serum was recovered by centrifugation. The specificity of the anti-TbC82 antibody was confirmed by Western blot analysis.

## Results

### C82 exhibits some distinctive features in trypanosomatids

**Figure 1.**
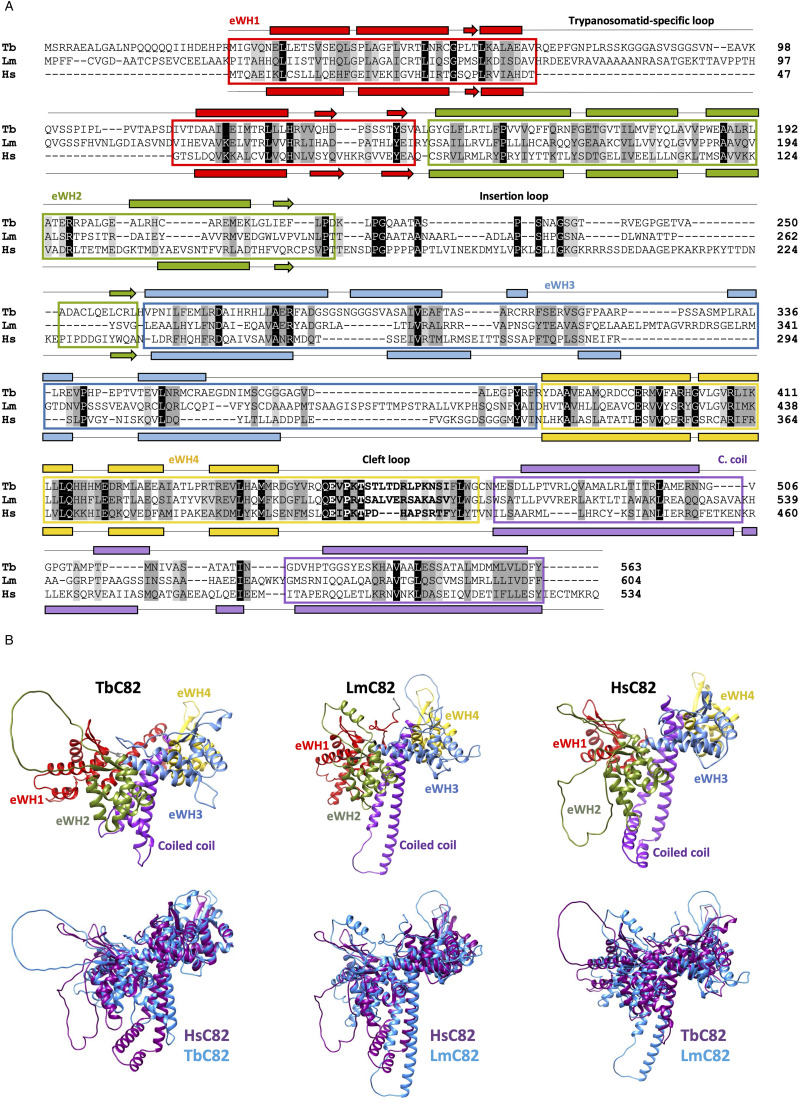
Sequence and predicted 3-dimensional structure analyses of C82 in *T. brucei* and *L. major*. (A) Alignment of the complete C82 amino acid sequences of *T. brucei* (Tb, Tb927.2.2990), *L. major* (Lm, LmjF27.2600) and *H. sapiens* (Hs, RPC62, NP_006459.3). The location of the 4 extended winged-helix (eWH) domains and the coiled-coil (C. coil) motif are indicated. Conserved residues are denoted by black shading, conserved substitutions by dark-grey shading and semiconserved substitutions by light-grey shading, according to the Clustal Ω program. The predicted secondary structure elements are shown for *T. brucei* (above the sequence) and *H. sapiens* (below the sequence). The *α*-helices are indicated with rectangles and the *β*-strands with arrows. (B) Predicted 3-dimensional structures of the entire TbC82 and LmC82 proteins generated with the AlphaFold program. For comparison, the structure of *H. sapiens* C82 (RPC62) is also presented. The structures are displayed in the same colours shown in panel A. Comparisons of the predicted structures with the iCn3D program revealed the following TM-scores: Tb/Lm = 0.740; Tb/Hs = 0.735; and Lm/Hs = 0.747.

The proteins encoded by Tb927.2.2990 and LmjF.27.2600 were identified as the C82 subunits of RNAP III in *T. brucei* (TbC82) and *L. major* (LmC82), respectively (Martínez-Calvillo *et al*., 2007). Sequence comparisons show that human C82 (RPC62) is 13.64% identical to TbC82 and 16.05% identical to LmC82 (Figs 1A and S1). This low sequence identity is not surprising, since the conservation of C82 orthologues is low across eukaryotes, with 19.25% identity between humans and *Saccharomyces cerevisiae*, and 25.78% identity between *Schizosaccharomyces pombe* and *S. cerevisiae* (Martínez-Calvillo *et al*., 2007) (Fig. S1). Regardless of the low sequence homology, both TbC82 and LmC82 contain the 4 characteristic eWH domains, and the coiled-coil domain in the C-terminal region (Fig. 1). While typical WH folds are composed of 3 *α*-helices and 3 *β*-strands in the order *α*1-*β*1-*α*2-*α*3-*β*2-*β*3, eWH domains in C82 possess an extra *α*-helix (*α*0) at the N terminus, the best fit being found for the eWH1 domain (Lefèvre *et al*., 2011) (Fig. 1A). However, a distinctive characteristic is that the insertion loop, which interrupts eWH2, is shorter in trypanosomatids (Fig. 1A). Moreover, these organisms contain an insertion within the eWH1 domain, which is not present in the other species analysed, and that we named ‘trypanosomatid-specific loop’ (Figs 1A and S1B).

As expected, C82 sequence conservation is higher among trypanosomatids, with identities ranging from 81.6 to 97.6% between *Leishmania* species, and from 57.8 to 80.2% between *Trypanosoma* species (Fig. S2). A 29.9% identity is observed between LmC82 and TbC82. To further analyse TbC82 and LmC82, their predicted 3-dimensional structure was generated with the AlphaFold program and compared to the structure of human C82. As shown in Fig. 1B, in all cases C82 is a globular protein mainly composed of *α*-helices, with 2 central long *α*-helices (the coiled-coil region) surrounded by the 4 well-defined eWH domains. Two clear differences are the presence of the longer insertion loop in the human eWH2, and the trypanosomatid-specific loop in the eWH1 of *T. brucei* and *L. major*. Notably, the predicted structure of TbC82 shows some differences when compared to the other 2 proteins and, consequently, the hypothetical structure of LmC82 is more similar to the human C82 structure than to the TbC82 structure (Fig. 1B).

### LmC82 and TbC82 localize to the nucleus

The subcellular localization of C82 was determined in *L. major* promastigotes and *T. brucei* procyclic forms by indirect immunofluorescence experiments. For that purpose, we produced cell lines where C82 was tagged with a carboxy-terminal PTP domain, which is composed of Protein A (Prot A) and Protein C (Prot C) epitopes separated by a TEV protease cleavage site (Schimanski *et al*., 2005). For *L. major* analysis, transgenic promastigotes that express the LmC82-PTP recombinant protein were produced with the pLmC82-PTP vector, obtained by cloning the complete LmC82 coding region into the episomal plasmid pB6-PTP (Moreno-Campos *et al*., 2016).

**Figure 2.**
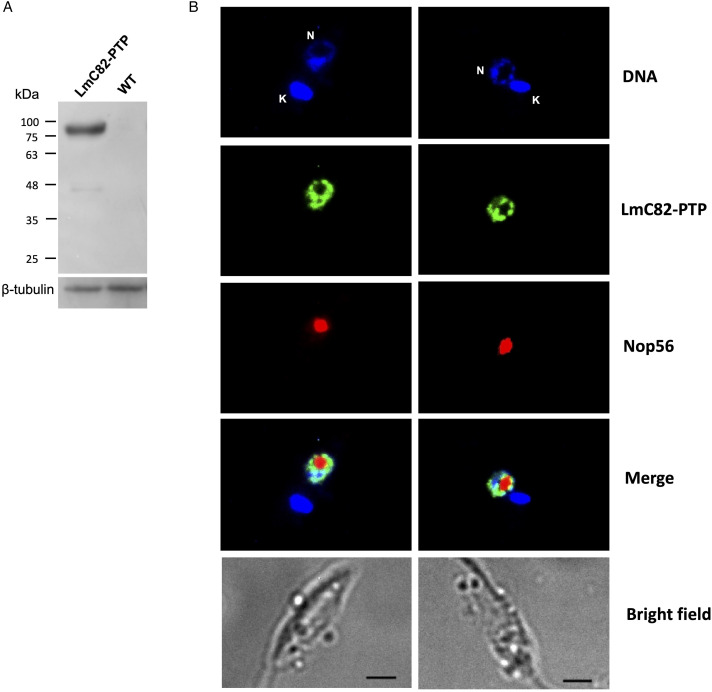
LmC82 is a nuclear protein. (A) Western blot analysis with parasites that express the LmC82-PTP protein and wild-type (WT) cells. Membranes were incubated with an antibody against Prot C and an anti-*β*-tubulin antibody (loading control). (B) Indirect immunofluorescence experiments to determine the subcellular localization of LmC82-PTP using an anti-Prot C antibody. An anti-LmNop56 antibody was used as a nucleolar marker. Parasites were then treated with a mixture of secondary anti-rabbit antibody conjugated with Alexa-Fluor 488 and anti-mouse antibody conjugated with Alexa Fluor 568 (Life Technologies Corporation). Nucleus (N) and kinetoplast (K) were stained with DAPI. Size bars represent 5 μm.

**Figure 3.**
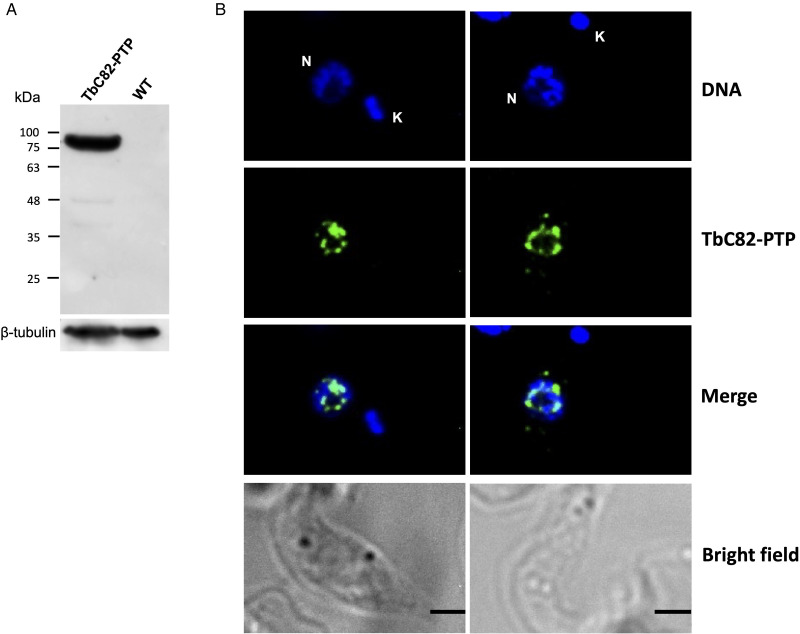
Subcellular localization of TbC82. (A) Western blot analysis with total protein from wild-type (WT) cells and parasites that express the TbC82-PTP protein using an anti-Prot C monoclonal antibody. As a loading control, *β*-tubulin was used. (B) The subcellular location of TbC82-PTP was analysed by indirect immunofluorescence assays using an anti-Prot C monoclonal antibody and an Alexa-Fluor 488 conjugated secondary antibody (Life Technologies Corporation). Nucleus (N) and kinetoplast (K) were stained with DAPI. Size bars represent 5 μm.

The expression of the LmC82-PTP protein was verified by Western blot experiments carried out with an anti-Prot C antibody, showing the expected band of ~83 kDa, product of the fusion of the 20 kDa PTP tag with the ~63 kDa LmC82 (Fig. 2A). Indirect immunofluorescence assays carried out with the anti-Prot C antibody showed that LmC82 is localized to the nucleus of the transfected parasites (Fig. 2B). While most signal was observed in the nucleoplasm, some fluorescence was found in the outer part of the nucleolus (Fig. 2B). In yeast, where 5S rRNA and tRNA genes are located within the nucleolus (Thompson *et al*., 2003), C82 is distributed throughout the entire nucleus (Wei *et al*., 2009). For *T. brucei*, the 3’-terminal coding sequence of TbC82 was cloned into the genome-integration vector pC-PTP-BLA (Schimanski *et al*., 2005) to obtain the plasmid pC-TbC82-PTP. Western blot experiments with the anti-Prot C antibody allowed the identification of the expected ~81 kDa band (the predicted mass of TbC82 is ~61 kDa) (Fig. 3A). Like in *L. major*, indirect immunofluorescence experiments showed that TbC82-PTP is a nuclear protein in *T. brucei* (Fig. 3B). This is in agreement with a genome-wide study that mapped the subcellular fate of most *T. brucei* proteins, where they found a nuclear localization for C-terminally mNeonGreen-tagged TbC82 (Billington *et al*., 2023) (data available on the TrypTag website, http://tryptag.org). Collectively, these results show that the PTP tag did not affect the nuclear localization of C82 in *L. major* and *T. brucei*.

### C82 is an essential protein in procyclic forms of *T. brucei*

**Figure 4.**
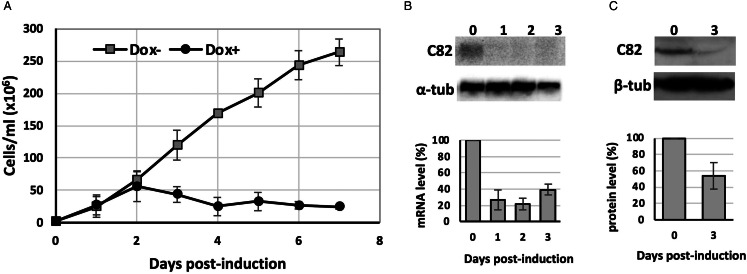
C82 is essential for cell growth of procyclic forms of *T. brucei*. (A) Growth curve of a clonal cell line obtained with the p2T7-TbC82 vector under non-induced (Dox−) and doxycycline-induced (Dox+) conditions. Cells were counted daily and diluted to a density of 2 × 10^6^ cells mL^−1^. The values represent the cumulative cell density multiplied by the dilution factor. Data points reflect the means of triplicate experiments. Standard deviation bars are shown. (B) Northern blot analysis of TbC82 mRNA in non-induced cells (0 days), and cells induced for 1, 2 or 3 days. The bands shown here and from 2 independent experiments were quantified and plotted, considering as 100% the RNA level obtained in the non-induced culture. Values represent means of the 3 experiments. Levels of TbC82 mRNA were normalized to the level of the *α*-tubulin mRNA (loading control). (C) Western blot analysis of the TbC82 protein in non-induced cells (0 days), and cells induced for 3 days using a specific anti-TbC82 polyclonal antibody. The bands shown here and from 2 independent experiments were quantified and plotted, considering as 100% the protein level obtained in the non-induced culture. Values represent means of the 3 experiments. Standard deviation bars are shown. TbC82 protein levels were normalized to the level of the *β*-tubulin protein (loading control).

**Figure 5.**
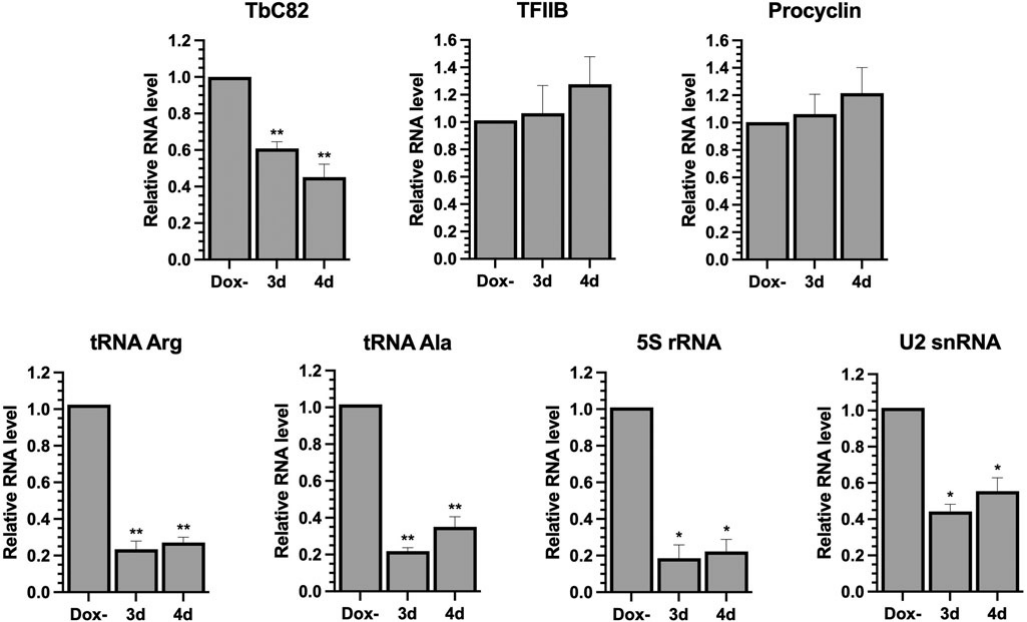
Depletion of TbC82 decreases the abundance of RNAP III-dependent transcripts. Quantitative PCR analysis of total RNA from induced (for 3 and 4 days) and non-induced (Dox−) TbC82 RNAi cultures. The RNAP III-dependent transcripts analysed were tRNA Arg, tRNA Ala, 5S rRNA and U2 snRNA. As controls, we analysed Procyclin (transcribed by RNAP I) and TFIIB (transcribed by RNAP II). We also evaluated the TbC82 transcript. Three biological replicates were analysed. All qPCR reactions were performed in duplicate, using primers and conditions that were optimized to produce a single amplicon of the correct size. Error bars indicate standard deviations. Statistically significant differences (Tukey's test) compared to the Dox-culture are indicated with * (*P* < 0.05) or ** (*P* < 0.01).

To assess whether TbC82 is indispensable for *T. brucei* survival, TbC82 was knocked down *in vivo* by RNAi. To that end, plasmid p2T7-C82 was generated by cloning a 419-bp fragment from the TbC82 coding region into p2T7-177, a vector that contains 2 opposite tetracycline-inducible T7 RNAP promoters to produce double-stranded RNA (Wickstead *et al*., 2002). Plasmid p2T7-C82 was transfected into the procyclic *T. brucei* cell line 29–13, which expresses the tetracycline repressor and T7 RNAP (Wirtz *et al*., 1999). The transfected population was cloned by limiting dilution, and a clonal cell line was selected for further analysis. To evaluate the effect of the TbC82 knock-down on the growth of *T. brucei*, cultures induced by the addition of the tetracycline analogue doxycycline (Dox+), and non-induced cultures (Dox–), were counted and diluted daily for 7 days. As shown in Fig. 4A, while Dox– parasites grew normally, Dox+ cells stopped growing 2 days after RNAi induction, leading to cell death 2 days later. To corroborate the TbC82 mRNA depletion after doxycycline induction, Northern blot analysis was performed, showing that the levels of the TbC82 mRNA were decreased by around 79% on day 2 post-induction (Fig. 4B). RT-qPCR experiments revealed that the levels of the TbC82 transcript were reduced by ~55% on day 4 post-induction (Fig. 5). Western blot analysis performed with a TbC82 polyclonal antiserum showed that the abundance of the TbC82 protein was reduced by approximately 48.5% after 3 days of induction (Fig. 4C). Thus, these results demonstrate that TbC82 is essential for the survival of procyclic forms of *T. brucei*.

### Knock-down of TbC82 led to a reduction in the levels of RNAP III-dependent transcripts

The effect of the ablation of TbC82 on the abundance of RNA molecules synthesized by RNAP III was analysed by RT-qPCR. These experiments were performed with total RNA isolated from cultures induced for 3 and 4 days. As shown in Fig. 5, a strong reduction in the abundance of all RNAP III-dependent transcripts analysed was observed, especially on day 3 post-induction. While the abundance of the tRNA-Arg and tRNA-Ala decreased to 23 and 21% of the control values, respectively, 5S rRNA was reduced to 18%. The level of the U2 snRNA was also reduced, but to a lesser extent (44% after 3 days of induction). Thus, these results demonstrate the participation of TbC82 in the transcription of all types of RNAP III-dependent genes in *T. brucei*. The abundances of the mRNAs encoding procyclin (synthesized by RNAP I) and TFIIB (transcribed by RNAP II) were not affected by TbC82 knock-down (Fig. 5).

### TbC82 and LmC82 associate with RNAP III-dependent genes

**Figure 6.**
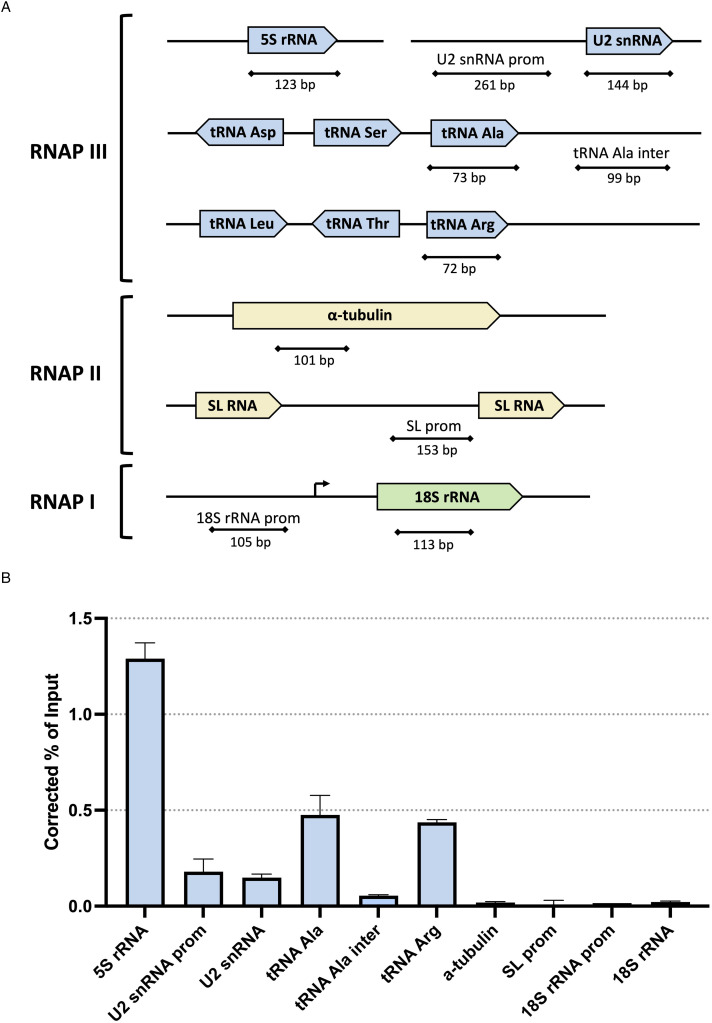
Chromatin immunoprecipitation analysis of TbC82. (A) Schematic drawing of the studied genes and amplicons quantified in panel B. Genomic regions transcribed by RNAP III, RNAP II and RNAP I are shown in blue, yellow and green, respectively. Maps are not shown to scale. (B) A ChIP grade anti-Prot A antibody was used to precipitate chromatin from the cell line that expresses the TbC82-PTP protein. Precipitated DNA was examined by qPCR. The results from 3 independent ChIP experiments, each analysing 2 qPCR reactions, are shown. Error bars indicate standard deviations. Results are presented as percentage of input, corrected by subtracting corresponding values from negative control precipitations performed with a nonspecific antiserum.

**Figure 7.**
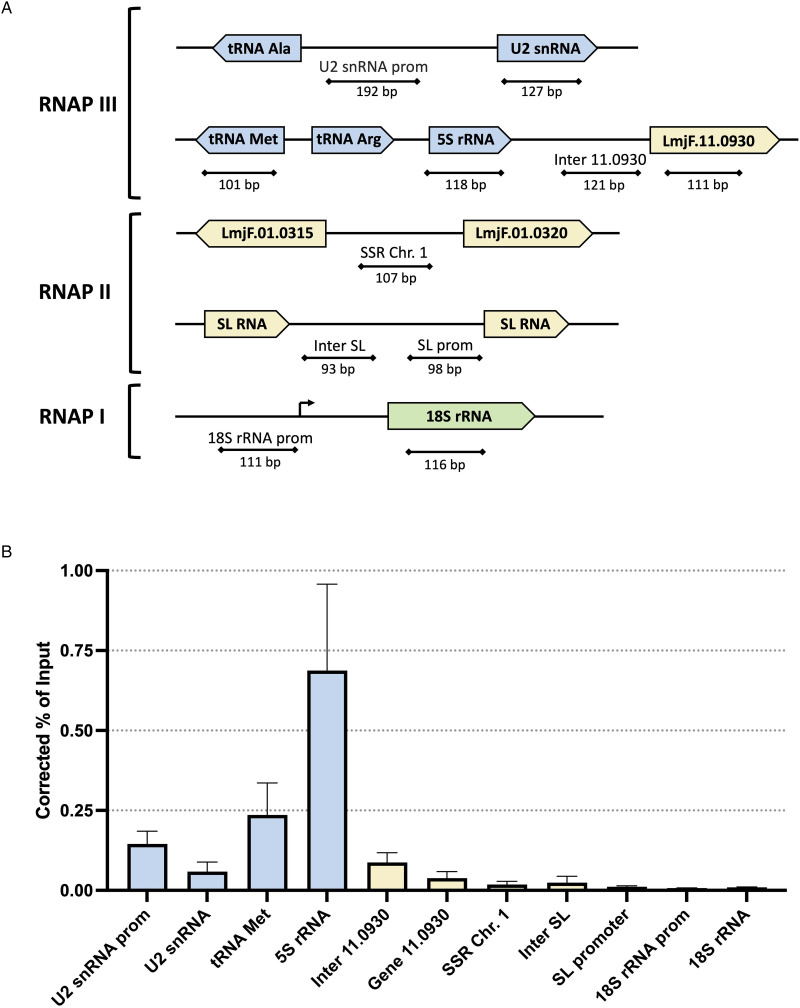
ChIP analysis of LmC82. (A) Genomic maps of the analysed loci. Regions transcribed by RNAP III, RNAP II and RNAP I are shown in blue, yellow and green, respectively. Maps are not shown to scale. (B) ChIP analysis using an anti-Prot A antibody was carried out with the cell line that expresses the LmC82-PTP protein. Precipitated DNA was examined by qPCR. The results from 3 independent ChIP experiments, each analysing 2 qPCR reactions, are shown. Error bars indicate standard deviations. Results are presented as percentage of input, corrected by subtracting corresponding values from negative control precipitations performed with a non-specific antiserum.

To demonstrate the *in vivo* binding of subunit C82 to genes transcribed by RNAP III in *T. brucei* and *L. major*, ChIP experiments were carried out with the clonal cell lines that express C82 fused to the PTP tag. Immunoprecipitations were performed with a ChIP-grade anti-Prot A antibody that recognizes the Prot A epitopes from the PTP tag, and with a non-specific mouse serum as a negative control. The association of TbC82-PTP and LmC82-PTP to the *T. brucei* and *L. major* genomes, respectively, was evaluated by qPCR assays conducted with the purified DNA. In *T. brucei*, strong occupancy of TbC82 was observed in the 5S rRNA, the tRNA-Arg and the tRNA-Ala genes (Fig. 6). The association of TbC82 was not found in an intergenic region located downstream of the tRNA-Ala gene. Enrichment of TbC82 was also detected in the U2 snRNA gene and its upstream promoter region. As anticipated, binding of TbC82 was not observed in the SL RNA promoter and the *α*-tubulin gene (transcribed by RNAP II) or in the 18S rRNA gene and its promoter region (transcribed by RNAP I) (Fig. 6). With *L. major* we obtained similar results, as high occupancy of LmC82 was found in the 5S rRNA and the tRNA-Met genes, as well as in the U2 snRNA gene and its upstream promoter region (Fig. 7). Binding of LmC82 was also observed in the intergenic region downstream of the 5S rRNA gene, but not in the neighbouring protein-coding gene LmjF.11.0930. Occupancy of LmC82 was not detected in the SSR from chromosome 1 and the SL RNA locus (transcribed by RNAP II), and the 18S rRNA gene and promoter region (transcribed by RNAP I) (Fig. 7). Altogether, these results show that C82 associates *in vivo* with RNAP III-dependent genes in *T. brucei* and *L. major*.

### Proteins that participate in RNAP III transcription and other functions copurified with TbC82-PTP and LmC82-PTP

**Figure 8.**
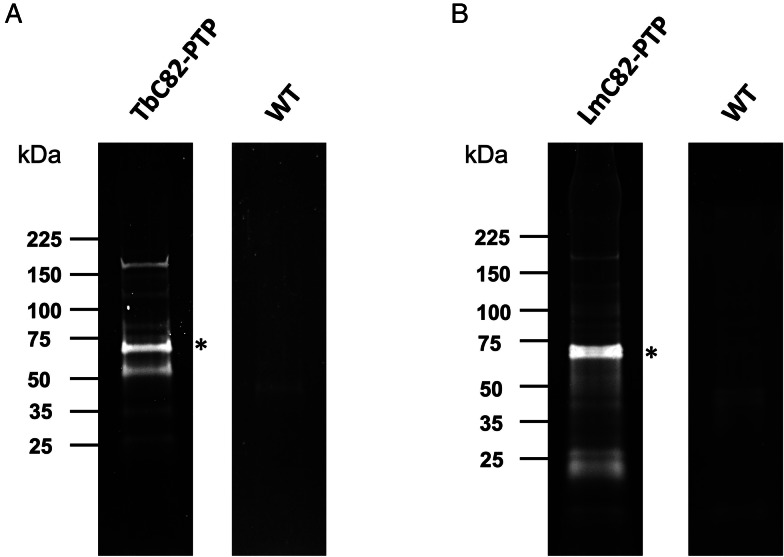
Tandem affinity purifications with *T. brucei* and *L. major* parasites expressing C82-PTP recombinant proteins. SDS-PAGE of proteins copurified with TbC82-PTP (A) and LmC82-PTP (B). The asterisks indicate the PTP-fused proteins. As controls, experiments with wild-type (WT) *T. brucei* (A) and *L. major* (B) parasites were also conducted. Proteins were analysed in 4–15% Mini- PROTEAN Precast Protein Gels (Bio-Rad) stained with SYPRO Ruby (Invitrogen).

In order to determine the proteins that interact directly or indirectly with the C82 subunit in *T. brucei* and *L. major*, tandem affinity purification experiments were carried out with the cell lines that express the recombinant proteins TbC82-PTP and LmC82-PTP, respectively. Associated proteins were isolated by IgG affinity chromatography, TEV protease elution and anti-Prot C affinity chromatography. SDS-PAGE analyses of the eluted material showed multiple protein bands, including 2 of ~64 and ~66 kDa that seem to correspond to the Prot C-tagged versions of TbC82 (Fig. 8A) and LmC82 (Fig. 8B), respectively (denoted with asterisks). As controls, purifications using wild-type *T. brucei* and *L. major* extracts were performed. Electrophoretic analysis of these controls showed the presence of a low number of faint bands (Fig. 8, WT lanes) that were identified by mass spectrometry as bovine serum albumin, human keratins, and several trypanosomatid ribosomal proteins, heat shock proteins, translation elongation factors, mitochondrial proteins, *α*- and *β*-tubulins and some other proteins (Table S1). These proteins are common contaminants in tandem affinity purifications (Mellacheruvu *et al*., 2013).

The TbC82-PTP and LmC82-PTP samples were examined by mass spectrometry and bioinformatic analyses, leading to the identification of numerous proteins (Table S1). To discriminate between contaminants and genuine C82 interactors, we applied the following criteria: the proteins identified on the wild-type cell purifications were considered contaminants, together with other proteins that we have usually detected in unrelated tandem affinity purifications (Florencio-Martínez *et al*., 2021; Mondragón-Rosas *et al*., 2024); also, proteins that presented an average coverage of less than 7.5% in *T. brucei*, or less than 20% in *L. major*, were regarded as possible contaminants. A lower coverage threshold was set for *T. brucei* because in this parasite we identified a smaller number of proteins, with lower overall protein coverages than those observed in *L. major* (Table S1). Accordingly, Tables 1 and 2 present the putative interacting partners of TbC82 and LmC82, respectively, which were grouped into 6 different categories: RNAP subunits, transcription factor TFIIIC subunits, putative transcription regulators, RNA-binding proteins, transport proteins and other functions. These proteins might associate directly or indirectly, via other proteins, with the C82 subunit. Since some DNA and RNA molecules could copurify with the tagged protein in affinity purifications, they may contribute to the indirect association of proteins with C82, and potentially influence the observed differences between *T. brucei* and *L. major* (see below).

**Table 1.** Proteins that copurified with TbC82^a^

TriTrypDB name	Protein name	Predicted size (kDa)	Peptides^b^	Coverage (%) ^c^
RNA polymerase subunits				
Tb927.2.2990	C82/RPC3 (RNAP III)	61.1	68, 52	61, 64
Tb927.3.3910	C34/RPC6 (RNAP III)	37.4	14, 13	33, 37
Tb927.11.3480	C37/RPC5 (RNAP III)	78.6	9, 6	15, 13
Tb927.10.15370	AC40/RPAC1 (RNAP I and III)	37.3	6, 6	19, 21
Tb927.3.2700	C17/RPC9 (RNAP III)	42.7	1, 3	3, 12
Transcription factor TFIIIC subunits				
Tb927.10.980	Tau95	67.4	32, 31	44, 49
Tb927.11.4520	Tau138	154.9	28, 30	24, 25
Tb927.1.3860	Tau131	131.4	23, 27	18, 20
Tb927.11.1590	Tau55	25.8	22, 20	69, 62
Putative transcription regulators				
Tb927.11.6310	Hypothetical protein (putative transcription regulator) *Trypanosoma*-specific	31.4	22, 18	50, 42
Tb927.11.10550	Hypothetical protein (putative transcription regulator)	90.5	14, 18	20, 24
Other functions				
Tb927.8.770	SUMO-interacting motif-containing protein	70.7	22, 18	44, 39
Tb927.1.4710	Hypothetical protein, conserved	19.5	4, 5	26, 35
Tb927.9.8880	Actin B	41.9	3, 4	11, 11
Tb927.10.13780	Glycogen synthase kinase 3 short	40.3	2, 3	7, 13
Tb927.10.15220	Hypothetical protein, conserved	22.1	1, 1	9, 9

a Proteins likely to be contaminants (including multiple ribosomal proteins, translation factors, tubulins, heat-shock proteins, mitochondrial proteins) were not included.

b Each digit indicates the number of peptides identified in 2 different tandem affinity purifications.

c Each number denotes the coverage found in 2 different experiments. Only proteins that show a coverage of at least 7.5%, in average, are shown.

**Table 2.** Putative interacting partners of LmC82^a^

TriTrypDB name	Protein name	Predicted size (kDa)	Peptides^b^	Coverage (%) ^c^
RNA polymerase subunits				
LmjF.27.2600	C82/RPC3 (RNAP III)	65.4	72, 87^d^	85, 81
LmjF.34.0360	C160/RPC1 (RNAP III)	173.6	40, 31	21, 27
LmjF.19.0660	AC40/RPAC1 (RNAP I and III)	47.4	28, 28	68, 69
LmjF.13.1370	C37/RPC5 (RNAP III)	76.5	23, 24	42, 41
LmjF.29.1320	C34/RPC6 (RNAP III)	48.1	19, 15	32, 43
LmjF.35.4170	C53/RPC4 (RNAP III)	37.9	16, 15	63, 63
LmjF.03.0790	C17/RPC9 (RNAP III)	45.4	16, 13	43, 45
LmjF.18.0780	RPB5 (RNAP II and III)	27.2	14, 15	72, 68
LmjF.19.0420	C11/RPC10 (RNAP III)	16.4	8, 4	51, 74
LmjF.28.2060	AC19/RPAC2 (RNAP I and III)	15.3	6, 5	77, 77
LmjF.25.1315	RPB10z (RNAP I)	9.7	2, 2	36, 36
Putative transcription regulators				
LmjF.18.0820	Hypothetical protein (probable TIP120)	289.8	41, 38	18, 20
LmjF.11.0950	Hypothetical protein (putative transcription regulator) *Leishmania*-specific	53	20, 21	42, 40
LmjF.35.0190	NLI interacting factor-like phosphatase, putative	27.1	3, 3	21, 19
RNA-binding proteins				
LmjF.21.0540	La protein	37.2	36, 30	62, 56
LmjF.36.5100	Hypothetical protein (probable RNA binding protein)	105.2	23, 29	43, 30
LmjF.22.1540	Alanyl-tRNA synthetase	106.3	19, 22	23, 22
LmjF.36.0050	PUF1	61.2	16, 17	36, 39
Transport proteins				
LmjF.33.1620	Hypothetical protein (probable Transportin 1)	101	25, 26	32, 32
LmjF.36.2510	Nucleoporin NUP96	96.9	16, 16	22, 22
LmjF.34.0490	Importin beta-1 subunit	95.6	16, 15	24, 25
LmjF.35.2330	Importin 1	99.4	15, 13	20, 22
LmjF.31.2980	Hypothetical protein (probable Importin)	105.8	14, 14	24, 21
Other functions				
LmjF.18.0700	HEAT repeats, putative	77.3	20, 13	33, 24
LmjF.04.1230	Actin	42	11, 12	40, 36
LmjF.07.0900	CAMK/CAMKL family protein kinase, putative	33.6	9, 8	30, 35
LmjF.10.0210	Nucleolar protein Nop56	52.7	8, 7	21, 20
LmjF.31.2960	Repressor of differentiation kinase 2	49.8	7, 8	22, 26
LmjF.36.5880	Ras-like small GTPases, putative	40.6	6, 6	23, 23
LmjF.32.1150	Hypothetical protein, conserved	33.5	5, 7	21, 32
LmjF.08.0940	Hypothetical protein, *Leishmania*-specific	28.8	4, 2	34, 22

a Proteins likely to be contaminants (including multiple ribosomal proteins, translation factors, tubulins, heat-shock proteins, mitochondrial proteins) were not included.

b Each digit indicates the number of peptides identified in 2 different tandem affinity purifications.

c Each number denotes the coverage found in 2 different experiments. Only proteins that show a coverage of at least 20%, in average, are shown.

d Around half of the identified peptides are shared with LmjF.020680, the smaller C82 isoform in *L. major*.

Regarding RNAPs, 4 RNAP III subunits (3 exclusive and 1 shared with RNAP I) copurified with TbC82, while 9 RNAP III subunits (either exclusive or shared with RNAP I or II), as well as an RNAP I subunit, were identified with LmC82. With TbC82, we also identified 4 TFIIIC subunits and 2 possible regulators of transcription, one of which is *Trypanosoma*-specific (Table 1). LmC82-PTP was copurified with a protein related to the TBP-Interacting Protein 120 (TIP120), the NLI interacting factor-like phosphatase, and a *Leishmania*-specific putative transcriptional regulator, together with several RNA-binding proteins and transport proteins (Table 2).

## Discussion

In this work, we studied the C82 subunit of RNAP III in *T. brucei* and *L. major*. To the best of our knowledge, this work represents the first characterization of C82 in any protozoan species. Although TbC82 and LmC82 show secondary and 3-dimensional structure conservation (Fig. 1), they (as well as C82 orthologues in other trypanosomatids) contain a trypanosomatid-specific loop within the eWH1 domain, whose size ranges from 30 amino acids (in *T. cruzi* and *T. rangeli*) to 49 amino acids in *Leishmania* spp. (Figs S1B and S2A). It is also worth noting that the insertion loop located in the eWH2 domain is shorter in trypanosomatids (only 12 amino acids in *Leptomonas seymouri* and *T. rangeli*) (Fig. S2A) than in other eukaryotes (64 amino acids in human cells) (Fig. 1). Thus, it would be interesting to determine the implications that these differences may have on the function of C82 and the complete RNAP III complex in trypanosomatids.

Interestingly, *L. major* possesses a truncated copy of C82 (LmjF.02.0680) (hereafter referred to as LmC82-short), not present in *Trypanosoma* spp., which seems to have arisen as the result of a recombination event between the ends of chromosomes 2 and 27, and that was considered a pseudogene (Martínez-Calvillo *et al*., 2007). A sequence comparison between LmC82 (604 amino acids) and LmC82-short (501 amino acids) showed that the latter lacks the eWH1 domain but contains a C-terminal extension (Fig. S3A). Also, LmC82-short presents a highly divergent eWH2 motif, but very conserved eWH3, eWH4 and coiled-coil domains (Fig. S3A). Mass spectrometry analyses of the LmC82-PTP complexes (see below) revealed that 47.8% of the identified peptides are exclusive to LmC82, and 52.1% are shared between both C82 isoforms. Interestingly, we identified a peptide exclusive to LmC82-short (Fig. S3A), indicating that this gene is expressed at the protein level. Since C82 has not been reported to form homodimers, a direct association between LmC82 and LmC82-short is unlikely. Thus, it is possible that LmC82-short interacts with other RNAP III subunits and that it was indirectly copurified in our experiments. Future studies will help to determine if LmC82-short is required for RNAP III transcription in *L. major*. Of note, a truncated C82 gene is found in the majority of the *Leishmania* species whose genome sequences are deposited on the TriTrypDB website. In all these species, the predicted protein sequences are even smaller than LmC82-short (for instance, 158 amino acids in *L. mexicana* and 192 amino acids in *L. donovani*) (Figs S3B and S3C), indicating that they could actually represent pseudogenes.

In yeast, the C82 subunit of RNAP III is essential for cell viability (Chiannilkulchai *et al*., 1992). Accordingly, ablation of C82 by RNAi demonstrated that this subunit is indispensable for the survival of procyclic forms of *T. brucei* (Fig. 4). It should be noted that previous genome-wide RNAi knock-down screens reported that TbC82 is not essential in the procyclic and bloodstream stages of the parasite (Alsford *et al*., 2011).

PTP-tag purifications with the TbC82 and LmC82 transgenic lines, followed by mass spectrometry and *in silico* analyses, led to the identification of multiple putative C82 interacting partners (Tables 1 and 2). While several RNAP III subunits were identified with both parasites, a RNAP I-specific subunit (RPB10z) was also copurified with LmC82. Among the RNAP III subunits, in both parasites we found C34, which in other species forms part of the RNAP III-specific heterotrimer (C82/C34/C31). In human cells, all 4 eWH domains from C82 (RPC62) seem to be required for the association with the C34 orthologue (RPC39) (Lefèvre *et al*., 2011; Li *et al*., 2021). It is worth mentioning that C82 and C34 are structurally related to subunits TFIIE*α* and TFIIE*β*, required for RNAP II transcription, and to archaeal TFE*α*/*β*, as they all contain multiple eWH motifs (Blombach *et al*., 2015; Khoo *et al*., 2018). Interestingly, we did not find an orthologue of the C31 subunit, the third component of the heterotrimer, and the only RNAP III subunit that has not been identified in trypanosomatids. In yeast and human cells, C31 possesses several conserved regions, including an Asp-Glu-rich acidic C-terminus, a pre-acidic domain, a conserved block and a stalk bridge helix (Boissier *et al*., 2015; Shekhar *et al*., 2023). None of the proteins that copurified with TbC82 and LmC82 seem to contain these domains, which suggests that C31 is absent in trypanosomatid parasites.

Remarkably, our results show that the degree of association between the TFIIIC subunits and C82 differs considerably between *T. brucei* and *L. major*. In the former, all 4 TFIIIC subunits that have been described in trypanosomatids (Mondragón-Rosas *et al*., 2024) copurified with TbC82 with a high number of peptides and coverage (Table 1). For instance, with the Tau95 subunit, both the number of identified peptides (32 and 31 in experiments 1 and 2, respectively) and the average coverage (46.5%) were even greater than those found with the C34 subunit of RNAP III (14 and 13 peptides; 35% average coverage). A similar result was observed with the Tau55 subunit (22 and 20 peptides; 65.5% coverage). In contrast, LmC82 was copurified with only 3 TFIIIC subunits, and they showed a low number of peptides and coverage (consequently, they are not shown in Table 2). The identified subunits are Tau131 (3 and 7 peptides; 5.5% coverage), Tau138 (3 and 3 peptides; 2.5% coverage) and Tau95 (1 and 4 peptides; 4.5% coverage). Similar to *T. brucei*, for C34 in *L. major* we identified 15 and 19 peptides with an average coverage of 37.5% (Table 2). In support of these findings, tandem affinity purifications performed with cells expressing the Tau95-PTP recombinant protein showed a robust association between Tau95 and C82 in *T. brucei*, but not in *L. major* (Mondragón-Rosas *et al*., 2024). Thus, altogether these results indicate that the interaction between the C82 subunit and transcription factor TFIIIC is strong in *T. brucei*, but weak in *L. major*. In human cells, a weak *in vitro* association between C82 (RPC62) and Tau95 (TFIIIC63) has been reported (Hsieh *et al*., 1999).

The robust copurification of all TFIIIC subunits with TbC82 is unexpected, taking into consideration that when RNAP subunits are used as baits in affinity purifications, the most abundant copurifying proteins are always other RNAP subunits (and not transcription factors), as reported in trypanosomatids (Walgraffe *et al*., 2005; Das *et al*., 2006; Devaux *et al*., 2006; Nguyen *et al*., 2006; Martínez-Calvillo *et al*., 2007) and other organisms (Jeronimo *et al*., 2004; Nguyen *et al*., 2015; Bhalla *et al*., 2019), and as found here for LmC82 (Table 2). As an example, tandem chromatin affinity purifications with yeast cells expressing a tagged C160 subunit resulted in the copious copurification of most RNAP III subunits, but TFIIIC was not identified (Nguyen *et al*., 2015). Thus, the atypical strong association between C82 and TFIIIC in *T. brucei* requires further analysis.

Other proteins that copurified with TbC82 are Tb927.11.6310 and Tb927.11.10550 (Table 1), which are annotated as hypothetical proteins. The former is a nuclear trypanosome-specific protein (Billington *et al*., 2023) that, according to the HHpred server, presents weak homology to some proteins involved in transcription regulation, such as mitochondrial transcription termination factor 2 (mTERF-2) (probability of 69%, *E*-value of 12) and elongation factor SPT5 (probability of 39%, *E*-value of 37). The DALI server identified Tb927.11.6310 as a feasible orthologue of a histone deacetylase Sir2-like protein (*z*-score of 2.5). Likewise, Tb927.11.10550 is a nuclear (and cytoplasmic) protein (Billington *et al*., 2023) that exhibits low sequence similarity to PF0610 (HHpred probability of 35%, *E*-value of 21), an archaeal protein presumably involved in transcription regulation (Wang *et al*., 2007), to the *α* subunit of TFIIE (HHpred probability of 20%, *E*-value of 59), and a subunit of the GATOR complex (*z*-score of 15.5), an upstream regulator of the mTORC1 pathway, which controls RNAP III transcription (Loissell-Baltazar and Dokudovskaya, 2021). In yeast, TORC1-dependent SUMOylation of C82 is required for RNAP III assembly and robust tRNA transcription (Chymkowitch *et al*., 2017). In this regard, among the proteins that copurified with TbC82 we found a SUMO-interacting motif-containing protein (Table 1), suggesting that C82 could also be SUMOylated in trypanosomatids.

With LmC82 also copurified a protein related to TIP120 (also known as CAND1) (HHpred probability of 100%, *E*-value of 1.4 × 10^−30^), which has been implicated in transcription activation of all 3 nuclear RNAPs in vertebrates (Makino *et al*., 1999). We also found an NLI-interacting factor-like phosphatase (Table 2) that is related to Fcp1, responsible for removing phosphates from the carboxy-terminal domain of the largest subunit of RNAP II (Hausmann and Shuman, 2003). Moreover, we identified LmjF.11.0950, a *Leishmania*-specific hypothetical protein that shows weak homology to mTERF-2 (HHpred probability of 42%, *E*-value of 30), to the SPT16 subunit of the FACT complex (HHpred probability of 23%, *E*-value of 100), and the alpha subunit of CTD kinase (*z*-score of 4.5).

In yeast, tRNA genes associate with nuclear pore complexes to coordinate RNAP III transcription with the nuclear export of tRNAs (Chen and Gartenberg, 2014). In that respect, several components of the nuclear pore complexes were identified with LmC82, such as nucleoporin NUP96, importin 1 and a putative transportin 1. Thus, as in yeast, RNAP III complexes might bind to nuclear pore proteins in *L. major*. Of note, no nuclear pore proteins were identified with TbC82. Among the RNA-binding proteins that copurified with LmC82, we found PUF1, involved in fine-tuning gene expression in trypanosomatids (Luu *et al*., 2006), as well as the La protein, which not only binds to RNA molecules transcribed by RNAP III, but also to mRNAs and other transcripts (Sommer and Heise, 2021). Actin copurified with both TbC82 and LmC82 (Tables 1 and 2). While it is a frequent contaminant in tandem affinity purifications, it has been demonstrated that human actin interacts with RNAP III to promote the synthesis of the U6 snRNA in a reconstituted *in vitro* transcription system (Hu *et al*., 2004). Therefore, actin may interact with C82 in trypanosomatids to control RNAP III activity.

It is important to point out that the C82-PTP protein was produced from an episome in *L. major*, which could result in the overproduction of the protein. In contrast, the C82 gene was PTP-tagged *in situ* in *T. brucei*. Nevertheless, previous works have shown comparable results with these 2 different approaches. For instance, the RNA editing complexes isolated in *L. tarentolae* (Aphasizhev *et al*., 2003) by overexpressing TAP-tagged proteins are very similar to those identified in *T. brucei* by *in situ* tagging (Schnaufer *et al*., 2003). Also, similar base J binding protein complexes have been reported in *L. major* (by overexpressing TAP-tagged proteins) (Jensen *et al*., 2021) and *T. brucei* (Kieft *et al*., 2020). Moreover, comparable CTR9 complexes were isolated in *L. major* (by episomal overexpression of tagged protein) (Jensen *et al*., 2021) and *T. brucei* (by *in situ* tagging) (Ouna *et al*., 2012). Thus, while overexpression of tagged proteins in *Leishmania* might potentially lead to the association with contaminants, the majority of the most abundant proteins represent real interactors; and non-specific proteins are also copurified in *T. brucei* by *in situ* tagging. Consequently, we believe that the differences we observed between *L. major* and *T. brucei* are genuine, and not the result of using different approaches to express the tagged C82 proteins.

In conclusion, in this work we have demonstrated that both TbC82 and LmC82 localize to the nucleus, where they associate *in vivo* with 5S rRNA, tRNA and U2 snRNA genes. Ablation of TbC82 led to a strong reduction in the levels of RNAP III-dependent transcripts and to the death of insect forms of *T. brucei*. The 2 isoforms of C82 present in *L. major* are expressed at the protein level and seem to be present in RNAP III complexes. Multiple putative TbC82 and LmC82 interactors were identified, including RNAP subunits such as C34. However, the orthologue of subunit C31 was not found in these parasites. Notably, our results suggest a robust association of C82 with TFIIIC in *T. brucei*, but not in *L. major*. Likewise, several components of the nuclear pore complexes copurified exclusively with LmC82. Moreover, novel putative regulators of transcription were identified in both parasites, some of which are genus-specific. Thus, our results indicate that RNAP III complexes are not identical in both parasites. The observed differences in the predicted 3-dimensional structures of TbC82 and LmC82 may be related, at least in part, to the distinctive interactions that they establish, which added to the presence of 2 C82 isoforms in *L. major*, most likely reflect differences in RNAP III transcription regulation between *T. brucei* and *L. major.* Interestingly, human cells possess 2 isoforms of subunit C31 (RPC7*α* and RPC7*β*) that give rise to 2 forms of RNAP III (RNAP III_A_ and RNAP III_B_). Differential expression of these 2 isoforms (and the RNAP III forms) has been observed during early embryogenesis, in differentiated cells, and during tumorigenesis (Cheng and Van Bortle, 2023). It would be interesting to explore whether different forms of RNAP III exist in *L. major*.

Several differences in the transcription process have been reported between *T. brucei* and *L. major*. For instance, RNAP I transcribes several protein-coding genes in *T. brucei*, but not in *Leishmania* (Günzl *et al*., 2003). Also, base J in *Leishmania* is required for proper transcription termination throughout the genome, but in *T. brucei* base J does not regulate transcription termination at most convergent SSRs (Reynolds *et al*., 2014). Moreover, retrotransposon hot spot proteins are trypanosome-specific factors (not found in *Leishmania*) that interact with RNAP II, and whose depletion impairs mRNA synthesis (Florini *et al*., 2019). Also, while the tRNA-Sec is transcribed by RNAP II in *T. brucei* (Aeby *et al*., 2010), it is synthesized by both RNAP II and RNAP III in *L. major* (Padilla-Mejía *et al*., 2015).

## Supporting information

Cano-Santiago et al. supplementary material 1 Cano-Santiago et al. supplementary material

Cano-Santiago et al. supplementary material 2 Cano-Santiago et al. supplementary material

## Data Availability

The mass spectrometry proteomics data have been deposited to the ProteomeXchange Consortium via the PRIDE partner repository (https://www.ebi.ac.uk/pride/) with the dataset identifiers PXD051017 and 10.6019/PXD051017.

## Supplementary material

The supplementary material for this article can be found at https://doi.org/10.1017/S0031182024000921.
