# Analyses of the essential C82 subunit uncovered some differences in RNA polymerase III transcription between *Trypanosoma brucei* and *Leishmania major* – CORRIGENDUM

**DOI:** 10.1017/S0031182026101954

**Published:** 2026-06

**Authors:** Andrés Cano-Santiago, Luis E. Florencio-Martínez, Daniel E. Vélez-Ramírez, Adrián J. Romero-Chaveste, Rebeca G. Manning-Cela, Tomás Nepomuceno-Mejía, Santiago Martínez-Calvillo

The authors regret the inclusion of an error in the above article. The sentence in the acknowledgement section that read:

‘This work is one of the requirements to obtain the PhD degree in Posgrado en Ciencias Biomédicas, Universidad Nacional Autónoma de México (UNAM), for José Andrés Cano Santiago’,

should have instead read:

‘This work is one of the requirements to obtain the PhD degree in Programa de Doctorado en Ciencias Biomédicas, Universidad Nacional Autónoma de México (UNAM), for José Andrés Cano Santiago’
