# Supplementary material for: Analyses of the essential C82 subunit uncovered some differences in RNA polymerase III transcription between Trypanosoma brucei and Leishmania major
Source: Parasitology. 2024 Nov 11;151(11):1185–200. doi: 10.1017/S0031182024000921 (PMC11894013; doi:10.1017/S0031182024000921)
Supplement: Cano-Santiago et al. supplementary material 1 — Cano-Santiago et al. supplementary material [file S0031182024000921sup001.pdf]

Supplementary Figures

Parasitology

Analyses of the essential C82 subunit uncovered some differences in RNA polymerase III transcription between *Trypanosoma brucei* and *Leishmania major*

Andrés Cano-Santiago, Luis E. Florencio-Martínez, Daniel E. Vélez-Ramírez, Adrián J. Romero-Chaveste, Rebeca G. Manning-Cela, Tomás Nepomuceno-Mejía and Santiago Martínez-Calvillo

Fig. S1. C82 sequence comparisons among eukaryotes. (A) Percent identities among amino acid sequences of C82. (B) Sequence alignment of the N-terminal region of C82. The trypanosomatid-specific loop is shown in bold letters. The sequences analyzed were: *T. brucei* (Tb, Tb927.2.2990), *L. major* (Lm, LmjF.27.2600), *Schizosaccharomyces pombe* (Sp, SPAPB1E7.03), *Saccharomyces cerevisiae* (Sc, NP_015516), *Homo sapiens* (Hs, NP_006459.3), *Mus musculus* (Mm, NP_083201), *Rattus norvegicus* (Rn, XP_008759532), *Arabidopsis thaliana* (At, NP_566914), *Caenorhabditis elegans* (Ce, NP_491807), *Plasmodium falciparum* (Pf, ETW47020.1), *Toxoplasma gondii* (Tg, XP_002366133), and *Naegleria fowleri* (Nf, KAF0973179).

Fig. S2. Sequence comparison of C82 in trypanosomatids. (A) C82 protein sequence alignment. (B) Percent identities among amino acid sequences of C82. The species analyzed were: *Leishmania braziliensis* (Lbr, LbrM.27.2720), *Leishmania tarentolae* (Lta, LtaP27.2650), *Leishmania mexicana* (Lmx, LmxM.27.2600), *Leishmania major* (Lmj, LmjF.27.2600), *Leishmania donovani* (Ld, LdCL_270033100), *Crithidia fasciculata* (Cfa, CFAC1_210027500), *Leptomonas seymouri* (Lse, Lsey_0405_0050), *Trypanosoma cruzi* (Tcr, TcCLB.509127.40), *Trypanosoma rangeli* (Tra, TRSC58_03156), *Trypanosoma brucei* (Tbr, Tb927.2.2990), and *Trypanosoma vivax* (Tvi, TvY486_0200650).

Fig. S3. Two isoforms of C82 are present in the *Leishmania* species. (A) Sequence comparison between C82 (LmjF.27.2600) and C82-short (LmjF. 02.0680) in *L. major*. The peptide specific to C82-short is indicated with the black bar at the boundaries of the eWH3 and eWH4 domains. (B) Sequence alignment of C82-short. (C) Percent identities among amino acid sequences of C82-short. The species analyzed were: *L. major* (Lmj, LmjF.02.0680), *L. aethiopica* (Lae, LAEL147_000034900), *L. arabica* (Lar, LARLEM1108_020011500), *L. braziliensis* (Lbr, LbrM.02.0660), *L. donovani* (Ldo, LdBPK_020650.1), *L. gerbilli* (Lgr, LGELEM452_020011500), *L. infantum* (Lin, LINF_020012100), *L. mexicana* (Lmx, LmxM.02.0680), *L. tropica* (LtrLTRL590_020012900), and *L. turanica* (Ltu, LTULEM423_020011100).

Fig. S1

A

	Tb<br>563 aa	Lm<br>604 aa	Sp<br>591 aa	Sc<br>654 aa	Hs<br>534 aa	Mm<br>533 aa	Rn<br>533 aa	At<br>523 aa	Ce<br>544 aa	Pf<br>915 aa	Tg<br>823 aa	Nf<br>611 aa
**Tb**	100	32.78	13.97	12.25	13.64	14.25	14.53	16.32	12.43	8.80	18.45	16.83
**Lm**		100	13.62	13.32	16.05	16.09	16.09	15.34	16.62	11.22	21.04	17.59
**Sp**			100	25.78	22.57	22.57	22.36	17.73	20.08	15.26	19.85	22.20
**Sc**				100	19.25	18.45	18.87	16.05	16.19	15.74	15.50	18.54
**Hs**					100	94.00	94.00	21.14	26.04	15.25	18.34	24.53
**Mm**						100	97.37	20.55	26.29	15.29	18.16	24.17
**Rn**							100	20.76	26.29	15.29	18.38	24.79
**At**								100	19.44	18.28	17.56	24.15
**Ce**									100	16.30	14.44	21.41
**Pf**										100	18.03	17.41
**Tg**											100	19.53
**Nf**												100

B

Trypanosomatid-specific loop

**Tb**	MS-RRAEAL-------------GALNPQQQQQIIHDEHPRMIGVQNELLETSVSEQLSPLAGFLVRTLNRCGP-LTLKALAEAV**RQEPFGNPLRSSKGGGASVSGGSVN--EAVKQ**	99
**Lm**	MP-FFCVGD----------------AATCPSEVCEELAAKPITAHHQLIISTVTHQLGPLAGAICRTLIQSGP-MSLKDISDAV**HRDEEVRAVAAAAANRASATGEKTTAVPPTHQ**	98
**Sp**	--MSQYAVELCEILVEEFFGDCCSAVASALLRHGR-LTIPMLQKR---------------------------------	42
**Sc**	M-DELLGEALSAENQTGESTVESEKLVTPEDVMTISSLEQRTLNPDLFLYKELVKAHLGERAASVIGMLVALGR-LSVRELVEK--------------------------------	82
**Hs**	-------------------------------------MTQAEIKLCSLLLQEHFGEIVEKIGVHLIRTGS-QPLRVIAHD------------------------------------	42
**Mm**	-------------------------------------MTQAEIKLCSLLLQEHFGEIVEKIGVHLVRTGS-QPLRVIAHD------------------------------------	42
**Rn**	-------------------------------------MTQAEIKLCSLLLQEHFGEIVEKIGVHLIRTGS-QPLRVIAHD------------------------------------	42
**At**	MSMSEFGIVYAIHIITVQFGSVVSKVCECLLRKGP-LSSRDISRLAESDI--	49
**Ce**	MGGGKYETQMCVIIIEDIFGKIVAKVMETILKESCQLSTLFYKLK--GQI--	48
**Pf**	MIKNELDYLKYIICDIFGSCCSDIVEIILLYGNKLSIYELINL--SSY--	46
**Tg**	MFRYEVQFACLLLEDAFGPVVSTCARLLLLHGS-LTILELRDLFLAVPSASGVSPPLSRCSRNAAAFA--SGCGRPSTHA------------------------------------	77
**Nf**	MN--KRTLALEILTDQFGDVIGKVGGVLMQFGP-QTMAEL-------------------------------------IQA------------------------------------	40

**Tb**	**VSSPIPL---PVTAPSD**IVTDAAIKEIMTRLLLHRVVQH----------------	135
**Lm**	**VGSSFHVNLGDIASVND**VIHEVAVKELVTRLVVHRLIHA----------------	137
**Sp**	----------------TSLPGPKIRQALVSLMQHHMVLYVTVIENVR-EVT----	76
**Sc**	---------------IDGMDVDSVKTTLVSLTQLRCVKYLQETAI----------	115
**Hs**	----------------TGTSLDQVKKALCVLVQHNLVSYQV-HKRGVVEYEAQCS	80
**Mm**	----------------TKASLDQVKKALCVLIHHNLVLYHV-HKRGVVEYEAQCS	80
**Rn**	----------------TKASLDQVKKALCVLIHHNLVIYHV-HKRGVVEYEAQCS	80
**At**	-------------------NHNKVKDILYLLIQHNCVQAFS----------IEPP	75
**Ce**	-------------------SNTLIRKSVSTLINFGFVTF-----------HLDTN	73
**Pf**	-------------------EFNVVRNVLLCLLIHNIVDVNIIYSKSIECNHLSPE	82
**Tg**	----------------GQDVYPLVRNALLVLLQHNLLLVTPIDGQGAPSLRAAPQ	116
**Nf**	----------------TSMQFPHIRNALLVLIQHNMCIYYDKNIYLQ-KLE----	74

Fig. S2

A

eWH1 — Trypanosomatid-specific loop

```
Lbr     MPLFRVSDAA---TRPSEVCEDLVAKPIAAHYQLIISTITDQLGPLAGAVCRALTQSGPMSLKDISDAVHRDEEVRAVAAAAANRASATGETTTAVPATHQLGSSFHVDLGDIASVNDVI   117
Lta     MPLFRVGDGA---TRPSEVCEDLAAKPITAHHQLIISTITHQLGPLAGAICRTLIQSGPMSLKEISDAVHRDEELRAVAAAAANRASAAGEKTTAVPPTHQVGSNFHVHLGDIASVNDVI   117
Lmx     MPLFCVGDVT---TRPSEVCEDLAAKPIAAHHQLIISTVTHQLGPLAGAICRILIQSGPMSLKDISDAVHRDEEVRAVAAAAANRASATGEKTTAVPPTHQVGSSFHVHLGDITCVNDVI   117
Lmj     MPFFCVGDAA---TCPSEVCEELAAKPITAHHQLIISTVTHQLGPLAGAICRTLIQSGPMSLKDISDAVHRDEEVRAVAAAAANRASATGEKTTAVPPTHQVGSSFHVNLGDIASVNDVI   117
Ldo     MPFFCVGDAA---TCPSEVCEDLAAKPITAHHQLIISTVTHQLGPLAGAICRTLIQSGPMSLKDISDAVHRDEEVRAVAAAAANRASATGEKTTAVPPTHQVGSSFHVNLGDIASVNDVI   117
Cfa     MPLFCVGSKA---TQPSEVCEDLSAKPIAAHHELLVTTVSHQLGPLAAAVCRALTHAGPMTLSDISDAVRRDEEVSGVAAAAANRASATGERTTAVPATHHVGTNLHVDVGELSAAKELI   117
Lse     MPLFCVGKAA---TQPSEVCEDLTAKPIAAHYELLVSTVAHQLGPLAGAVCRALTHTGPMTLMDLSEAVRRDEDVRGVAAAAASHASATGETTTAVPPTHRIGSNLHVDLGELSSANELI   117
Tcr     ------------MTRRHDGHHDTHARILTEHQELLESSISEQLGPLAGFLVHALNRRGPLTLKEVADAVKREPFNNPLHTG--------------DATKHIGCPLAVPV---TA-GDMA   89
Tra     -----------MARVWHDSHHDEHARMLTEHAELLESSVSEQLGPLAGFLVRTLNRRGALTLRGLADAVKREPFGNPLSTG--------------DAARHVGCPLAVSA---VA-GDVG   90
Tbr     MSRRAEALGALNPQQQQQIIHDEHPRMIGVQNELLETSVSEQLSPLAGFLVRTLNRCGPLTLKALAEAVRQEPFGNPLRSSKGGGASVSGGS--VNEAVKQVSSPIPLPV---TAPSDIV   115
Tvi     MVQRSVSDAASRNQRRQSQSVSNSPCVVTAHNELLESSIARQLGPLAEFLVRTLNRCGPLTLKELADTLKRESFNNPLRSGNDGET----------DATKHTRSPIELPV---TVGADVA   107
                          .   .     :  : :*: :::: **.***  : : * : * ::*  ::::::::     : :.                .::    : :        ::
```

eWH2

```
Lbr     HEVAVKELVTRLVVHRLVHTDPVTHLYEMRYGSAILLRVLFPLLLHCARQRYGEAAKCVLLVAYQLGVVPPRAAVQVALSRTPSLTHEAIEYAVVRLVEDGWLVPVPNLPTAAPDAATPA   237
Lta     HEVAVKELVTRLVVHRLIHADPITHLYELRHGSAILLRVLFPLLLHCARQQYGEAAKCVLLVVYQLGVVPPRAAVQVALSRTPSITRDAIEYAVVRMVENGWLVPVLKSPSAAHGAATV-   236
Lmx     HEVAVKELVTRLVVHRLIHADPATHLYEIRYGSAILLRVLFPLLLHCARQQYGEAAKCVLLVVYQLGVVPPRAAVQVALSRTPSITRDAIEYAVVRMVEDGWLVPVHNSPTAAPGAATAA   237
Lmj     HEVAVKELVTRLVVHRLIHADPATHLYEIRYGSAILLRVLFPLLLHCARQQYGEAAKCVLLVVYQLGVVPPRAAVQVALSRTPSITRDAIEYAVVRMVEDGWLVPVLNLPTTAPGAATAA   237
Ldo     HEVAVKELVTRLVVHRLIHADTATHLYEIRYGSAILLRVLFPLLLHCARQQYGEAAKCVLLVVYQLGVVPPRAAVQVALSRTPSITRDAIEYAVVRMAEDGWLVPVLNSPTAAPGATTAA   237
Cfa     HEVAVKEVVTRLVLHRLVYADPARQLYGMRLGSAMLLRVFFPLLLHCVRQQHGEAAKCVLLAVYQLGAVPPVAAVQVALSRTPSIGRDALEYAVTQLVAHGWLLPVAVNSND--------   229
Lse     HEVAVKEVVTRLVLHRLVYADPSTQLYGMRLGSALLLRVLFPLLVHCVRQQYGEAAKCVLLAVYQLGAVPPVAAVQVALSRTPSIGRDALDYAVTQLAADGWLVPVMVDGAN--------   229
Tcr     TDAAIKEIMARLVLHRVVCYDTENHVYSVALGYGLFLRTLFPLVMQFFQRRYGETGVTLLMVFYQLATVPRDAAVRLAMERRPALSEA-LRHCAHEMERLGFIEYFSAGGSAEAAA----   204
Tra     TDAAIKELMARLVLHRVVWYDAERHVYGVALGYGLLLRTLFPLVMQFFQRRYGETGVTLLMVFHQLAMVPWEAALRLAMERRPALSEA-LRHCGREMERLGFIEFVSAGKSVEVAV----   205
Tbr     TDAAIKEIMTRLLLHRVVQHDPSSSTYSVALGYGLFLRTLFPVVVQFFQRNFGETGVTILMVFYQLAVVPWEAALRLATERRPALGEA-LRHCAREMEKLGLIEFLPDKLPGQAATASPS   234
Tvi     ADAAIKEVVTRLLLHRVVRYDAESQTYSVSLGYGLFLRTLFPLVVQLFHRRYGEAGVTILMVYYQLAVIPLEQALRLAIERRPTLSEA-LRRCAQEMEVLGLVEPLHAPLAKETHA--PT   224
         :.*:**:::**::**::  *     * :  * .::**.:**::::  ::..**:.  :*:. :**. :*    *:::* .* *:: .   :   .  .:    * :  .
```

eWH3 — Insertion loop

```
Lbr     ---LRADGASSHLGGSLDLWSATRPHCVGLQAALHCLFSDAIE-QAVAERYADG-------RLALTLVRALRRRTAPNSEYTEAVASFQELAAELPPTA-----GVRRDRSGEP---RVG   338
Lta     --TRAADVASSHPDDNLDLWNATTPYCVGLEAALHYLFNDAIE-QAVAERYADG-------RLALTLIRTLRRRAAPNSGYTEASASFQELAAELPTTA-----GVRRDHSGEP---RMG   338
Lmx     NAARLADLAPSHPGGNADLWNAATPYCVGLEAALHYLFNDAIE-QAVAERYADG-------RLALTLVRVLRRRVAPNSGYTEAVASFQELAAELPTTA-----GVRRDRSGEP---LMG   341
Lmj     NAARLADLAPSHPGSNADLWNATTPYSVGLEAALHYLFNDAIE-QAVAERYADG-------RLALTLVRALRRRVAPNSGYTEAVASFQELAAELPMTA-----GVRRDRSGEL---RMG   341
Ldo     NAARLADLAPSHPGGNADMWNTTTPYSVGLEAALHYLFNDAIE-QAVAERYADG-------RLALTLVRALRRRIAPNSGYTEAVASFQELAAELPTTA-----GVRRDRSGEP---RMG   341
Cfa     --------AAVKESSAEDVWDAATPYRIGLESALFSLMNDALE-QAVAERFADG-------GLALTLVRALRRRAAPNSRYTESVASWQELAAELPTHASVGGFGVRRDRDGKP---VAL   330
Lse     --------PTS-SCDVADVPSATTPYCIGLESAIFFLMGDALE-QTVAERHADG-------GLALTLVRALRRRAAPNSQYGDGAASWQELAAELPTPA-----GVRRDRSGQP---VQL   324
Tcr     -----------TT-TTHSTGLSPDPCRLHVGNILAEMLHDAIQQQLLAERFADG-------GVAAAVMEAFTTAT-RTRRLVERSH---------------GFPPVHPRTSASLPLRVL   288
Tra     -----------T--ATPTATLSPDPCRLHVGNILAEMLHDAIQHQLLAERFADG-------GVAAAVMEAFATLG-RGRRLEERSP---------------MFPPVHPRASVSLPLRVL   288
Tbr     NAGSGTRVEGPGE-TVAADACLQELCRLHVPNILFEMLRDAIHRHLLAERFADGSGSNGGGSVASAIVEAFTASA-RCRRFSERVS---------------GFPAARPPSSASMPLRAL   336
Tvi     EAPSRQST---TT-ANSLALPSLDPCQLHVGNILAEMLREAIHRQLLMERFADASGNS--SSVAVGIVDCFTKAT-RTRHLKERLD---------------GFPPMHPRVSPPVSLRAL   321
                                    : :   :  :: :*:. : : **.**.         :*  ::  :            :                           :   .
```

```
Lbr     TDSVSSGSAEAVRRCLERLCQPVALFA-GCNAAAPMTPAAGSS-----------PNFTTMPSTRALLVKPQSESDLYALDDVTAVQLLQEAVCERVVYSRYGVLGVRIMRLLLQHHFLEE   446
Lta     TDNLPSSSVEAVQRCLQRLCQPIVL-Y-SCYATASTTSATGVS-----------PSLTMMPSTRALLVKPHSQSNVYSMDHVAAVHLLQEAVCERVVYSRYGVLGVRIMKLLLQHHFLEE   445
Lmx     TDNVPSSSVEALQRCLQRLCQPIVF-Y-SCDAAAPMTSVAGIS-----------PSFTTMPSTRALLVKPHSQSNFYAIDHVTAVHLLQETVCERVVYSRYGVLGVRIMKLLLQHHFLEE   448
Lmj     TDNVPSSSVEAVQRCLQRLCQPIVF-Y-SCDAAAPMTSAAGIS-----------PSFTTMPSTRALLVKPHSQSNFYAIDHVTAVHLLQEAVCERVVYSRYGVLGVRIMKLLLQHHFLEE   448
Ldo     TDNVPSSSVEAVQRCLQRLCQPIVF-Y-SCDAAAPMTSAAGIS-----------PSFTTMPSTRALLVKPHSQSNFYAIDHVTAVHLLQEAVCERVVYSRYGVLGVRIMKLLLQHHFLEE   448
Cfa     PET--TQSAAAVQRCLQRLCQPIVM-L-SCDAAASLTATTPLSPPGAATASSSSAASFPLPATRALLVQPNNQRGFYALDDVTATHLLQEAVCERVVYTGYGVLGVRIMKLLLQHRFLEE   446
Lse     PEN--TRSAEAVQRCLERLCQPIVL-L-SCDGANSSTAAAAFH----ASASSSSTLPVALPATRALLVQPHKQHGFYALDSVTAVHLLQEAVCERVVYTGYGVLGVRIMKLLLQHHYLEE   436
Tcr     LREVP-MAEGVVVEAVSRMCRPDGDSLLSNDGTAT-VA-----------------------------------IEGPYRFRYDAAIEAMQRDCCERLVFARHGVLGVRLMKLLLQHHNMED   372
Tra     LREVP-LAESAVMATVSRMCRPDGDSLLSNDGTAT-VA-----------------------------------IEGPYRFRYDAAIDAMRRDCCERLVFARHGVLGVRLMKLLLQHHNMED   372
Tbr     LREVP-HPEPTVTEVLNRMCRAEGDNIMSCGGGAGVDA-----------------------------------LEGPYRFRYDAAVEAMQRDCCERMVFARHGVLGVRLIKLLLQHHHMED   421
Tvi     LCEVP-HDEQAVLETLSRMCRSDGDFLVCSDGSASSGS-----------------------------------MDGSFSFQYHSAVEAMRRDCCERFVFARHGVLGLRLIKLLLQHHNMED   406
                  .:    :.*:*:             .                                        . : :  :* . ::.   ***.*:: :****:*:::*****: :*:
```

eWH4 — Cleft loop — C. coil

```
Lbr     RTLAEQSVATYVKAREVLHRMFKDGFLLQQEVPRTSALVERSAKASVYLWGLSWSATLLPAVRERLAKTLTIAWVKLREAQQQASAVAKPAA----CV--------RSSPAASGGIKSSA   554
Lta     RTLAEQSVATYVKVREVLHQMFKDGFLLQQEVPRTSALVERSAKASVYLWGLNWSGTLLPVVRERLAKTLTIAWVKLREAQQQASAVPKPAS----RV--------GSTPTAGGSFNSSV   553
Lmx     RTLAEQSVATYVKVREVLHQMFKDGFLLQQEVPRTSALVERPAKASVYLWGLSWSATLLPVVRERLAKTLTIAWEKLREAQQQASAVAKPAA----GV--------HPTPAAGSGINSSA   556
Lmj     RTLAEQSIATYVKVREVLHQMFKDGFLLQQEVPRTSALVERSAKASVYLWGLSWSATLLPVVRERLAKTLTIAWAKLREAQQQASAVAKHAA----GG--------RPTPAAGSSINSSA   556
Ldo     RTLAEQSIATYVKVREVLHQMFKDGFLLQQEVPRTSALVERSAKASVYLWGLSWSATLLPVVRERLAKTLTIAWAKLREAQQQASAVAKPAA----GV--------RPTPAAGSSINSSA   556
Cfa     RTLAEQSVATYTKTRDVLHRMFKDGFLSQQEVPRSGALAERAAKGSIFLWGLSWSGTLLPTVRERLAKTLCIAWAKLQQHQQQQQLAAALASESARL---RPTP-SSSPAVGGGDAAAV   562
Lse     RTLAEQSVATYAKTRDVLHRMFKDGFLTQQEVPRSGALADRTAKGSIFLWGLSWAATLLPAVRERLAKTLSIAWVKLREAQGQQAAAASPIASQPT-----------------NGEPASR   539
Tcr     RMLAEEAIATLSRTREVLHAMLRDGYVRQQEVPKSGVMADRQPKNSVFLWGCSMEKDLLPAVRMQVAKALRISLARLSVERQTATSKVASFTTTTTTGSNNNNNHHNNCCAASSTAATSV   492
Tra     RMLAEEAIATLPRTREVLHAMLRDGYVRQQEVPKSGVMADRQPKNSVFLWGCSLEKDLLPVVRMQVAKALRLTLARLSLERQAAASQAASFTAAAATAGGAIP------------AAASK   480
Tbr     RMLAEEAIATLPRTREVLHAMMRDGYVRQQEVPKTSTLTDRLPKNSIFLWGCNMESDLLPTVRLQVAMALRLTITRLAMERNNGVGPGTAMPTPMNI----------------------   518
Tvi     RMLAEEAIATLPRTREVLHAMMRDGYVRQQEVPKTGTLADRLPKNSVFLWSCNLHRELLPTIRVQVATALRMTLARLRIEREEGANSAQPATSPLGI----------------------   503
        * ***:::**  :.*:*** *::**:: *****::..:..:*  * *::**. .    ***.:* ::* :* ::  :*   :
```

C. coil

```
Lbr     AHAEEVEAQWKSGMSRSIQQALQTQRTITGLQSCVMSLMRLLLIVDFF   602
Lta     AHTEEVEAQWKYGMSGNIQQALQAQRTVTGLQSCVMSLMRLLLIVDFF   601
Lmx     AHAEEIEAQWKYGMSRNIQQVLQAQRAVTGLQSCVMSLMRLLLIVDFF   604
Lmj     AHAEEIEAQWKYGMSRNIQQALQAQRAVTGLQSCVMSLMRLLLIVDFF   604
Ldo     AHAEEIEAQWKYGMSRNIQQALQAQRAVTGLQSCVMSLMRLLLIVDFF   604
Cfa     SVMEDAEAQWRHGLTRSTQQALQAQRTVTGLQSCVMSIMRLLLIVDFF   610
Lse     QAADDAEAMRKYGLTRRMQQALRVQRTVTGLQSCVMSLTRLLVVVDFF   587
Tcr     AAGLSLTAGIRGDTGEVIAETLRCQRAVAALESSVSALMGTMLVLDFY   540
Tra     VATSEAAAGVRRPTGEATAEALRPQRAVAALESSALALMGTMLVLDFY   528
Tbr     VASATATIN---GDVHPTGGSYESKHAVAALESSATALMDMMLVLDFY   563
Tvi     VAAHSSVSG---VNSQGAKVNDGVNYPILALESSASALMEMMLVLDFF   548
                          :  : .*:*.. ::   ::::**:
```

B

	Lbr	Lta	Lmx	Lmj	Ldo	Cfa	Lse	Tcr	Tra	Tbr	Tvi
**Lbr**	100	81.6	84	84.3	84.3	66.7	68.2	33.6	31.9	28.7	31.1
**Lta**		100	88.1	88.5	88.8	67	67	33.3	32.8	29.3	30.2
**Lmx**			100	94.8	95.2	68.4	68.5	32.9	32.2	29.2	29.9
**Lmj**				100	97.6	68.2	68.5	33.5	33.6	29.9	30.4
**Ldo**					100	68.1	69.1	33.9	33.7	29.7	30.8
**Cfa**						100	77.3	34.2	35.4	31.9	29.9
**Lse**							100	34.1	34.1	31.4	31.5
**Tcr**								100	80.2	61.8	60.7
**Tra**									100	61.4	58.9
**Tbr**										100	57.8
**Tvi**											100

Fig. S3

A

```
                                         eWH1                                  Trypanosomatid-specific loop
LmjF.27.2600   MPFFCVGDAATCPSEVCEELAAKPITAHHQLIISTVTHQLGPLAGAICRTLIQSGPMSLKDISDAVHRDEEVRAVAAAAANRASATGEKTTAVPPTHQVG   100
LmjF.02.0680   ----------------------------------------------------------------------------------------------------   0

                                                                                              eWH2
LmjF.27.2600   SSFHVNLGDIASVNDVIHEVAVKELVTRLVVHRLIHADPATHLYEIRYGSAILLRVLFPLLLHCARQQYGE-AAKCVLLVVYQLGVVPPRAAVQVALSRT   199
LmjF.02.0680   ----------------------------------------------MGAIIMVLRERVRASEEYIRRLEGTAHSACLEAVLNRLMEVARA----------   44

                                    Insertion loop
LmjF.27.2600   PSITRDAIEYAVVRMVEDGWLVPVLNLPTT-APGAATAANAARLADLAPSHPGSN-ADL-----WNATTPYSVGLEAALHYLFNDAIEQAVAERYADGRL   292
LmjF.02.0680   --HCRETTRMRTWDALRQVWEGTDIAASEELHCGLIN--PRRRTRGRSPASPPPCSTRMHARRRRRSDTACTSSLSKCHQKVERMDTPQAVAERYADGRL   140

                                       eWH3
LmjF.27.2600   ALTLVRALRRRVAPNSGYTEAVASFQELAAELPMTAGVRRDRSGELRMGTDNVPSSSVEAVQRCLQRLCQPIVFYSCDAAAPMTSAAGISPSFTTMPSTR   392
LmjF.02.0680   GLTFVRALRRRIAPASGYTEA----------------VRRDRSGEPRMGTDNVPSSSVEAVQRCLQRLCQPIVFYRCDAAAPMSSAAGISPSFTTMPSTR   224

                                                         eWH4                                 Cleft loop
LmjF.27.2600   ALLVKPHSQSNFYAIDHVTAVHLLQEAVCERVVYSRYGVLGVRIMKLLLQHHFLEERTLAEQSIATYVKVREVLHQMFKDGFLLQQEVPRTSALVERSAK   492
LmjF.02.0680   ALLVKPHSQSNFYAVDHVTAVHLLQEAVCERVVHSRYGVLGVRIMKLLLQHHFLEERTLAEQSIATYVKVREVLHQMFKDGFLLQQEVPRTSALVERSAK   324

                    C. coil                                                               C. coil
LmjF.27.2600   ASVYLWGLSWSATLLPVVRERLAKTLTIAWAKLREAQQQASAVAKHAAGGRPTPAAGSSINSSAAHAEEIEAQWKYGMSRNIQQALQAQRAVTGLQSCVM   592
LmjF.02.0680   ASVYLWGLSWSATLLPVVRERLAKTLTIAWAKLREAQQQASAVAKHAAGGRPTPAAGSSINSSAAHAEEIEAQWKYGMSRNIQQALQAQRAVTGLQSCVM   424

LmjF.27.2600   SLMRLLLIVDFF-----------------------------------------------------------------   604
LmjF.02.0680   SLMRLLLIVDFFFFKGVRRTVERQTSVGVVGVASLSLSPYRCFGCHARQGDGAVRPARRPSPLFSPSCAPARVVRGA   501
```

B

eWH2 — Insertion loop

**Lmj**	MGAIIMVLRERVRASEEYIRRLEGTAHSACLEAVLNRLMEVARAHCRETTRMRTWDALRQVWEGTDIAASEELHCGLINPRRRTRGRSPASPPPCS-TRM	99
**Lae**	--	0
**Lar**	--MTRMRTWDALRQVWEGMDIAASEELHCGLINPRRRTRGRSSASPPPRSTCGM	52
**Lbr**	--MNR-------------------------------	3
**Ldo**	--	0
**Lgr**	--	0
**Lin**	--	0
**Lmx**	--	0
**Ltr**	MGPLIMVLREGVRVPVEYIRGLEGPAHSACLEAVLNRLMEVARARCRETKPMRTWDALRRVWEGMDIAASEELHCGLINPRRRTRGRSSASPPPRSTCGM	100
**Ltu**	MDPIIMVLRERVRVSEEYIRRLEGPAHSACLEAVLNRLMEVARARCRETTRMRTWDALRQVWEGMDIAASEELHCGLINPQRRTHGRSSASPPPRSTCGM	100

eWH3

**Lmj**	HARRRRRSDTACTSSLSKCHQKVERMDTPQAVAERYADGRL-------------GLTFVRALRRRIAP-ASGYTEAVRRDRSGEPRMGTDNVPSSSVEAV	185
**Lae**	----------------------MHRRPRPS----AVQTDGF-------------PLTLVRALRRCIAP-SSGYTEAVRRDRSGELRMGTDNVPSSSVEAV	60
**Lar**	HARRRRRADTACTSSLSKCHQNVERMDTPQAAAERYADGRL-------------ALTLVRALRRCIAP-DSGYTEAVRRDRSGEPRTGTDNVPSSSAEAV	138
**Lbr**	--------------------------SADQHVAAKYPDKRRVRTSPHPSQGREIKTHMRRRLRGRGREGGDDTGQVADGECGGEPRVGTDSVSSGSAEAV	77
**Ldo**	--MGTDNVPSSSVEAV	14
**Lgr**	--	0
**Lin**	--MGTDNVPSSSVEAV	14
**Lmx**	--MGTDNVSSSSVEAV	14
**Ltr**	HARRRRRAGTACTSSLSKCHRKVERMDAPQAAAERYADGRL-------------ALTLVRALRRRVAP-NSGYTEAVRRDRSGELRMGTDNVPSSSVVAV	186
**Ltu**	HARRRRRADTACTSSLSKCHQKVERMDTPQAAAERYADGRL-------------ALTLVRALRRRVAP-DSGYTEAVRRDRSGEPRMGTDNVPSSSVEAV	186

eWH4

**Lmj**	QRCLQRLCQPIV-FYRCDAAAPMSSAAGISPSFTTMPSTRALLVKPHSQSNFYAVDHVTAVHLLQEAVCERVVHSRYGVLGVRIMKLLLQHHFLEERTLA	284
**Lae**	QRCLQRLCQPIV-FYSCDAAAPMTSAAGISPSFTTMPSTRALLVKPHSQSNFYAIDHVTAVHLLQEAVCERVVYSRYGVLGVRIMKLLLQHHFLEERTLA	159
**Lar**	QRCLQRLCQSIV-FYSCDAAAPMTSAAGISQSFTTMPSTRALLVKPHSQSNFYAIDHVTAVHLLQEAVCERVVYSRYGVLGVCIMKLLLQHHFLEERTLA	237
**Lbr**	RRCLERLCQPVALFAGCNAAAPMTPAAGSSPNFTTMPSTRALFVKPQSESDLYALDDVTAVQLLQEAVCERVVYSRYGVLGVRIMRLLLQHHFLEERTLA	177
**Ldo**	QRCLQRLCQPIV-FYSCDTAAPMTSAAGISPSFTTMPSTRALLVKPHSQSNFYAIDHVTAVHLLQEAVCERVVYSRYGVLGVRIMKLLLQHHFLEERTLA	113
**Lgr**	--MKLLLQHHFLEERTLA	16
**Lin**	QRCLQRLCQPIV-FYSCDAAAPMTSAAGISPSFTTMPSTRALLVKPHSQSNFYAIDHVTAVHLLQEAVCERVVYSRYGVLGVRIMKLLLQHHFLEERTLA	113
**Lmx**	----QPLCQTVV-FYSCDAAAPMTSAAGISPSFTTMPSTRALLVKPHSQSNFYAIDHVTAVHLLQEAVCERVVYSRYGVLGVCIMKLLLQHHFLEERTLA	109
**Ltr**	QRCLQRLCQPIV-FV---	200
**Ltu**	QRCLQRLCQPIV-FYSCDPAAPMTSAAGISPSFTTMPSTRALLVKPHSQSNFYAIDHVTAVHLLQEAVCERVVYSRYGVLGVRIMKLLLQHHFLGERTLA	285

Cleft loop — C. coil

**Lmj**	EQSIATYVKVREVLHQMFKDGFLLQQ**EVPRTSALVERSAKASV**YLWGLSWSATLLPVVRERLAKTLTIAWAKLREAQQQASAVAKHAAGGRPTPAAGSSI	384
**Lae**	EQSIATYVKVREVLHQMFKDGFLLQQEVPRTSALVERSAKASVYLWGLSWSATLLPVVRERLAKTLTIAWAKLREAQQQASAVAKPAAGVRPTPAAGSSI	259
**Lar**	EQSIATYVEVREVLHQMFKDGFLLQQGVPRTSALVERSAKASVYLWGLSWSATLLPVVRERLAKTLTIAWAKLREAQQQASAVAKPAAGVRPTPAAGSSI	337
**Lbr**	EQSVATYVKAREVLHRMFKDGFLLQQEVPRTSALVERSAKASVYLWGLSWSATLLPAVRERLAKTLTIAWVKLREAQQQASAVAKPAACVRSSPAASGGI	277
**Ldo**	EQSIATYVKVREVLHQMFKDGFLLQQEVPRTSALVERSAKASVYLWGLSWSATLLPVVRERLAKTLTIAWAKLREAQQH---------------------	192
**Lgr**	EQSIATYVKVREVLHQMFKDGFLLQQEVPRTSALVERSAKASVYLWGLSWSATLLPVVRERLAKALTIAWAKLREAQQQASAVAKPAAGVRPTPAAGSSI	116
**Lin**	EQSIATYVKVREVLHQMFKDGFLLQQEVPRTSALVERSAKASVYLWGLSWSATLLPVVRERLAKTLTIAWAKLREAQQH---------------------	192
**Lmx**	EQSVATYVKVREVLHQMFKDGFLLQQEVPRTSALVERPAKASVYLWGGG---	158
**Ltr**	--	200
**Ltu**	EQSIATYVKAREVLHQMFKDGFLLQQEVPRTSALVERSAKASVYLWGLSWSATLLPVVRERLAKTLTIAWAKLREAQLQASAVAKPAAGVRPTPAAGSSI	385

C. coil

**Lmj**	NSSAAHAEEIEAQWKYGMSRNIQQALQAQRAVTGLQSCVMSLMRLLLIVDFFFFKGVRRTVERQTSVGVVGVASLSLSPYRCFGCHARQGDGAVRPARRP	484
**Lae**	NSSAAHAEEIEAQWKYGMSRNIQQALQAQRAVTGLQSCVMSLMRLLLIVDFF--	311
**Lar**	NSSAAHAEEIEAQWKYGMSRNIQQALQAQRAVTGLQSCVMSLMRLLLIVDFF--	389
**Lbr**	KSSAAHAEEVEAQWKSGMSRSIQQALQTQRTITGLQSCVMSLMRLLLIVDFF--	329
**Ldo**	--	192
**Lgr**	NSSAAHAEEIEAQWKYGISRNIQQALQAQRAVTGLQSCVMSLMRLLLIVDFF--	168
**Lin**	--	192
**Lmx**	--	158
**Ltr**	--	200
**Ltu**	NSSAAHAEEIEAQWKYGMSRNIPQALQAQRAVTGLQSCVMSLMRLLLIVVFF--	437

**Lmj**	SPLFSPSCAPARVVRGA	501
**Lae**	-----------------	311
**Lar**	-----------------	389
**Lbr**	-----------------	329
**Ldo**	-----------------	192
**Lgr**	-----------------	168
**Lin**	-----------------	192
**Lmx**	-----------------	158
**Ltr**	-----------------	200
**Ltu**	-----------------	437

C

	**Lmj** 501 aa	**Lae** 311 aa	**Lar** 389 aa	**Lbr** 329 aa	**Ldo** 192 aa	**Lgr** 168 aa	**Lin** 192 aa	**Lmx** 158 aa	**Ltr** 200 aa	**Ltu** 437 aa
**Lmj**	100	92.28	93.30	74.20	96.88	97.62	97.40	91.77	85.43	93.12
**Lae**		100	92.60	74.59	98.96	98.81	99.48	94.30	74.32	92.28
**Lar**			100	73.57	95.31	97.62	95.83	91.77	88.82	95.12
**Lbr**				100	83.85	87.50	84.38	81.65	38.96	73.89
**Ldo**					100	97.89	99.48	93.67	92.86	97.40
**Lgr**						100	97.89	93.85	24.41	95.83
**Lin**							100	94.30	92.86	97.40
**Lmx**								100	75.00	92.41
**Ltr**									100	91.00
**Ltu**										100
